# Paced Breathing Associated With Pupil Diameter Oscillations at the Same Rate and Reduced Lapses in Attention

**DOI:** 10.1111/psyp.70003

**Published:** 2025-02-04

**Authors:** Ralph Andrews, Michael Melnychuk, Sarah Moran, Teigan Walsh, Sophie Boylan, Paul Dockree

**Affiliations:** ^1^ Trinity College Dublin, Trinity Institute of Neuroscience (TCIN) Dublin Ireland

**Keywords:** oscillations, paced breathing, pupil diameter, pupillometry, respiration, sustained attention

## Abstract

A dynamical systems model proposes that respiratory, locus coeruleus—noradrenaline (LC‐NA), and cortical attentional systems interact, producing emergent states of attention. We tested a prediction that fixing respiratory pace (versus spontaneous respiration) stabilizes oscillations in pupil diameter (LC‐NA proxy) and attentional state. Primary comparisons were between ‘Instructed Breath’ (IB) and ‘No Instructed Breath’ (NIB) groups. Secondarily, we investigated the effects of shifting respiratory frequency in the IB group from 0.15 to 0.1–0.15 Hz in Experiment 1 (*n* = 55) and 0.15–0.1 Hz only in Experiment 2 (*n* = 48) (replication). In the Paced Auditory Cue Entrainment (PACE) task, participants heard two auditory tones, alternating higher and lower pitches, cycling continuously. Tones acted as a breath guide for IB and an attention monitor for both groups. Participants gave rhythmic mouse responses to the transition points between tones (left for high‐to‐low, right for low‐to‐high). We derived accuracy of mouse click timing (RTm), variability in click timing (RTVL), and counts of erroneously inverting the left/right rhythm (IRs and Switches). Despite no differences between groups in RTm or RTVL, IB committed significantly fewer IRs and switches, indicating less lapses in attention during paced breathing. Differences in behavioral metrics were present across tone cycle frequencies but not exclusive to IB, so breath frequency did not appear to have a specific effect. Pupil diameter oscillations in IB closely tracked the frequency of the instructed breathing, implicating LC‐NA activity as being entrained by the breath intervention. We conclude that pacing respiratory frequency did stabilize attention, possibly through stabilizing fluctuations in LC‐NA.

## Impact statement

Slow‐paced breathing is core to meditative practices, yet direct tests of an effect on attention are lacking. We addressed this using a novel task and testing for a novel mechanism of mediation. This further advances recent research that has drawn links between respiratory and cognitive dynamics.

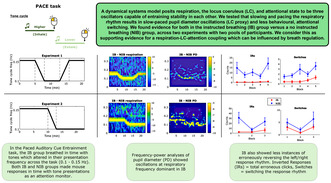

## 1 Introduction

The breath is a central tool utilized by yogis and meditators of other faiths to stabilize attention. Within the eight limbs of yoga, the 4th limb is ‘Pranayama’ or ‘breath suspension/regulation,’ and it is a key preparation for the 6th limb, ‘Dharana’ or ‘concentration.’ (Yoga Sutras of Patanjali, circa 200 BCE). There are parallels drawn here between slowing and stabilizing the breath and slowing and stabilizing the fluctuations of the mind.

Despite this ancient and widely held practice, a link between slow‐paced breathing and stable attention has been remarkably understudied in contemporary Western science. Research on slow‐paced breathing (< 10 breaths/min) has largely focused on the reliably induced mental relaxation and improved emotional regulation. Both of which are thought to be mediated via vagal nerve activity and changes in heart rate variability (Zaccaro et al. 2018). These pathways may provide an indirect mechanism for facilitating stable attention through parasympathetic‐mediated relaxation; however, the yogic texts are clear in the directness of this relationship, and recent neuroscientific evidence suggests likewise.

A surge of interest in respiration within cognitive neuroscience has shown that in humans, respiration modulates electroencephalography (EEG) (Herrero et al. 2018; Perl et al. 2019; Zelano et al. 2016) and magnetoencephalography (MEG) (Kluger and Gross 2021) activity and further, respiratory phase (i.e., inhale‐exhale) modulates task performance (Johannknecht and Kayser 2022; Nakamura et al. 2022; Perl et al. 2019; Zelano et al. 2016) and perception (Flexman, Demaree, and Simpson 1974; Grund et al. 2022; Kluger et al. 2021) across a range of cognitive domains. In these studies, there was no breath intervention, and these effects, along with respiratory entrainment to task events in some cases, were present from a single session. Thus, these findings appear to represent an innate quality of respiration and its interaction with brain and mind.

This work has led to theories that posit the breath as a low‐frequency ‘pacemaker rhythm’ that can shape the rhythm of information processing in the brain (Boyadzhieva and Kayhan 2021; Brændholt et al. 2023; Goheen et al. 2024), possibly in part due to the evolutionary and developmental primacy of the olfactory sense and the breath that drives it (Perl et al. 2019). If respiration does indeed significantly and continuously influence the pace of cognitive rhythms, it follows that slowing down and stabilizing respiration should slow down and stabilize fluctuations in attentional state.

The first research group to formally propose a mechanism for this link came from Melnychuk et al. (2018) who posit the locus coeruleus‐noradrenaline (LC‐NA) system as a key nexus that could facilitate coupling between respiration and attention. Changes in attentional state over time are concomitant to the activity of functional networks; for example, there is thought to be an antagonistic relationship between the goal‐driven, externally focused nature of the dorsal attentional network and the mind‐wandering, introspective‐related default mode network (Fox et al. 2005, 2015). Melnychuk et al.'s model extends this idea of intra‐cortical competition driving attentional changes by proposing that respiration is having an additional influence, from the periphery, through the LC.

Research on mice has shown that a key inspiratory driving nucleus, the preBötzinger complex, directly projects to the LC and that ablation of these neurons modulates arousal state (Yackle et al. 2017). Further, a considerable number of LC neurons are CO_2_ sensitive in mice and rats, responding in a dose‐dependent manner to hypercapnia (Gargaglioni, Hartzler, and Putnam 2010) and relaying this information to other key respiratory areas (Krohn et al. 2023; Lopes et al. 2016).

With respect to attention, the LC‐NA system has been central to a seminal model by Aston‐Jones and Cohen that considers the widespread connectivity and modulatory properties of the LC‐NA system (Samuels and Szabadi 2008) of being able to facilitate the allocation of attention by determining the neural gain of attentional networks (Aston‐Jones and Cohen 2005b, 2005a). The LC displays two distinct firing modes: (i) tonic mode, where baseline NA firing is high, cortical neural gain is high, and network activation and attentional state are distributed, and (ii) phasic mode, where NA firing is task‐locked, neural gain is low, task‐specific networks are active, and attention is selective. A fluctuating dominance in these modes facilitates an optimal exploration‐exploitation balance of behavior, periodically broadening and narrowing the scope of focus. Much of this model is evidenced by animal research (Aston‐Jones and Cohen 2005b); however, recent studies in humans using pupil diameter (PD) as an LC proxy measure have supported the model also (Jepma and Nieuwenhuis 2011; Murphy et al. 2011; Regnath and Mathôt 2021; van den Brink, Murphy, and Nieuwenhuis 2016).

Pupil diameter is a commonly used, reliable proxy measure for LC‐NA activity (Bang et al. 2023; DiNuzzo et al. 2019; Elman et al. 2017; Meissner et al. 2023; Murphy et al. 2014), and we take advantage of this relationship in the present study. However, it should be noted that this relationship is not fully defined, as changes in PD also correlate to other brain areas (DiNuzzo et al. 2019; Joshi et al. 2016), and there are other neuromodulators of PD besides NA (Cazettes et al. 2021; Larsen and Waters 2018; Reimer et al. 2016).

The dynamical systems model from Melnychuk et al. (2018) proposes that if the LC is simultaneously interacting with oscillatory respiratory information and cortically mediated oscillations in attentional state, modulating either respiration or top‐down attentional control could influence the activity of the other.

Respiration, LC‐NA, and cortical attention network activities can be thought of as ‘non‐linear, autonomous, noisy oscillators.’ Here we can take respiration as an example to briefly explain these terms within a dynamical systems framework. Respiratory activity is non‐linear, since its underlying dynamics rely on non‐linear processes. For example, the threshold response of neural activity in the preBötzinger complex, which generates respiratory rhythm, is non‐linear. Autonomous means that it is self‐driven and independent of the time factor. Respiration is driven by intrinsic feedback loops predominantly within the brainstem and does not depend on time or external factors for its drive. Finally, respiration is noisy due to the inherent variability in neural activity, mechanical properties, sensory feedback, and environmental influences.

Melnychuk et al. (2018, 2021) provided evidence that these non‐linear, autonomous, noisy oscillators exhibit coupled relationships, i.e., they can interact with each other and synchronize activity. They demonstrated phase synchronization between respiration and LC functional magnetic resonance imaging (fMRI) blood‐oxygen‐level‐dependent (BOLD) activity as well as PD (Melnychuk et al. 2018). In a later study they extended this relationship to a significant synchronization and information transfer (Granger causality) between respiration, PD, and EEG‐derived frontal theta: beta ratio as an attention‐related cortical measure (Melnychuk et al. 2021). They reason these oscillators could act coherently, pushing attention into a stable attractor state, or incoherently, destabilizing attention. Stable attractor states are states that the dynamical system tends towards due to, e.g., phase synchronization between the coupled oscillators. With regards to attention, this could be a familiar emergent state such as internally‐directed planning or externally‐directed listening. Direct tests of the model's predictions in humans during breath manipulation are yet to be studied.

The present study aims to build upon the foundational theory and evidence for the respiration‐LC‐attention coupling model by testing one of its predictions. Namely, that stabilizing and reducing respiratory frequency should reduce variability in LC‐NA firing, assayed from PD activity, and also reduce behavioral variability through stabilization of attentional state.

Considerable work has sought to investigate whether respiration can modulate pupil activity, independent of research on attention. A recent review on the topic (Schaefer et al. 2023) concluded that the strength of evidence for an effect of respiratory phase on pupil dynamics was “low” and of rate and depth was “very low.” Direct tests of a link between respiration rate and pupil dynamics have so far generally sought to correlate their respective activities at rest. To our knowledge, only one study has attempted to manipulate respiration rate for a modulating effect on PD activity (Daum and Fry 1981). They found support for such an effect, however only with three participants. Therefore, as well as primarily acting as a proxy measure to ascertain LC‐NA activity, we sought to test for direct evidence within this field of respiration‐pupil modulation.

In the present study we sought to test the hypothesis that slow‐paced breathing leads to a stabilization of attention concomitant with a reduction in PD oscillation frequency. To this end, we implemented a novel task, the Paced Auditory Cue Entrainment (PACE) task, where the stimuli are both a breath guide and rhythmic response targets for monitoring behavior‐derived stability of attention during the breath intervention. PD was monitored continuously as a proxy for LC‐NA activity. We chose a respiratory frequency range of 0.1–0.15 Hz to align with the current literature on psychophysiological effects of slow‐paced breathing (Zaccaro et al. 2018) and the range for slow pranayama. We attained behavioral metrics of attention. Results were compared within the ‘Instructed Breath’ (IB) group to test for an effect of respiration frequency and against a ‘No Instructed Breath’ (NIB) control group to test for an effect of guided, paced breathing.

Comprehensively, our study questions were:

Does slow‐paced breathing:

- Enhance behavioral attentional stability?
- Stabilize PD oscillatory frequency?
- Affect the phase‐coupling relationship between respiration and PD?

## 2 Methods

### 2.1 Participants

Participants were recruited through Trinity College Dublin communications, all students at the college, aged 18–29 years old. Inclusion criteria for the study required participants not currently experiencing respiratory illness and not undertaking regular breathwork exercises. In Experiment 1, 66 participants attended data collection (36 female), and in Experiment 2, 63 (32 female). These were two completely distinct groups of participants with no overlap across experiments. The number of participants included in each analysis will be stated in each section, as it depended on data quality and/or task compliance. We based our required sample size on previous studies investigating a modulatory effect of respiratory rate on PD, as there has been more work here than for an effect on attention. Sample sizes under 15 have shown positive findings, whereas those over 15 have been negative (Schaefer et al. 2023; their Figure 4).

### 2.2 Protocol

Participants were given an information sheet that described the study broadly as an investigation into the physiological correlates of sustained attention. Before the main task, the Paced Auditory Cue Entrainment (PACE) task, resting respiration rate was recorded for 3 min. Participants were randomly assigned to an experimental ‘Instructed Breath’ (IB) group or a control ‘No Instructed Breath’ (NIB) group using the MATLAB randi() function to provide an array of ‘1’ or ‘2’, the length of the intended participant pool. Participants wore headphones to hear the task's auditory stimuli, and volume was kept constant across participants. We attempted to keep the NIB group naive to our focus on respiration by informing them that the respiration belt was also a heart rate monitor and that the study was about ‘the physiological response to periods of sustained attention.’ Responses to the post‐study qualitative questionnaire indicated that NIB participants did not think the study had a focus on respiration (see Appendix S1, Experiment 1). Both groups completed the main task with the presence (IB) or absence (NIB) of breathing instructions. They then filled in a post‐task qualitative questionnaire, were debriefed, and paid €10 (TangoCard voucher) or given course credits for the hour session.

### 2.3 Paced Auditory Cue Entrainment (PACE) Task

**FIGURE 1:**
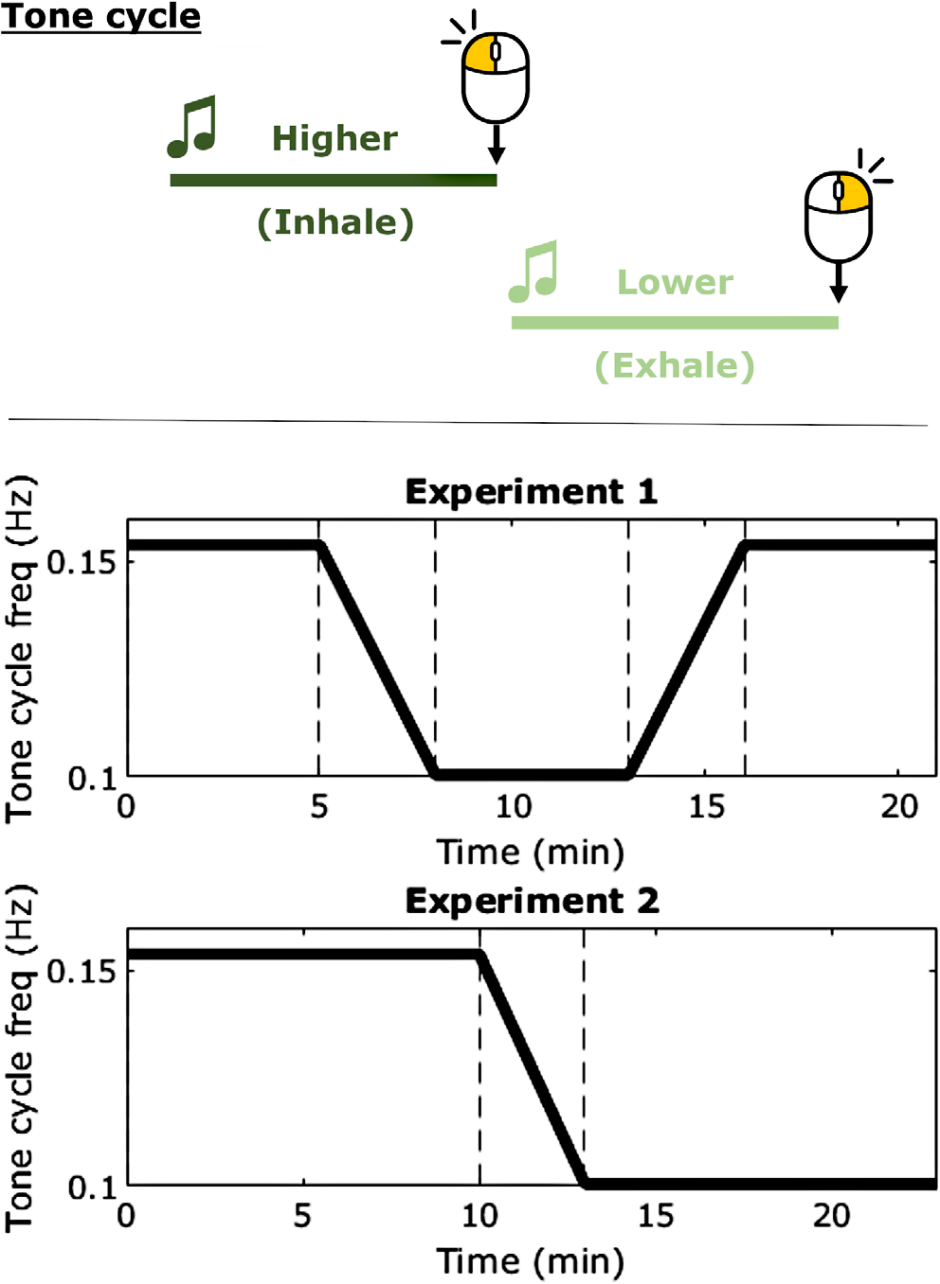
Schematic for Paced Auditory Cue Entrainment (PACE) task. Participants heard a higher‐pitched tone and lower‐pitched tone cycling continuously. Participants were instructed to rhythmically respond to the stimuli transition points with mouse clicks so that left clicks landed on the high → low transition and right clicks on the low → high. An ‘Instructed Breath’ (IB) group had to additionally inhale during the higher tone and exhale during the lower tone. A ‘No Instructed Breath’ (NIB) group only responded with mouse clicks. The tone cycle frequency changed over time. In Experiment 1, the tone cycle frequency shifts from 0.15 to 0.1 Hz and back again. In Experiment 2, there is only a shift from 0.15 Hz slowing to 0.1 Hz. The rate of required mouse responses and the rate of respiration for IB only, changed accordingly.

The PACE task was novelly designed in PsychoPy to be a simultaneous ‘paced breath guide with changing rates’ and a ‘sustained attention task’; see Figure 1 for the task schematic. In this study, two distinct experiments were conducted using the PACE task. In Experiment 1, the task lasts 21 min, where the participant hears two tones continuously alternating, one higher and one lower in pitch. We based this breath guide format of alternating pitched tones on online breath guides as they seemed intuitive to follow for breathwork‐naïve participants. There are five blocks: In block 1 (5 mins), the two tones alternate in a 6.5 s cycle, i.e., 3.25 s higher tone, 3.25 s lower tone, or 0.15 Hz. In block 2 (3 mins), the cycle incrementally increases in length each cycle until it reaches 9.95 s, or 0.1 Hz. Block 3 (5 mins) is a constant 0.1 Hz cycle. Block 4 (3 mins): The tone cycle decreases in length back to 0.15 Hz. Block 5 (5 mins) is a constant 0.15 Hz cycle (same as Block 1). In Experiment 2, Block 1 is 10 mins of 0.15 Hz, Block 2 is 3 mins of frequency slowing, and Block 3 is 10 mins of 0.1 Hz, totaling 23 mins.

All participants are instructed to time rhythmic mouse clicks precisely to the transition points between tones: left click for the high → low transition (HL) and right click for the low → high transition (LH).

The IB group was given additional instructions to breathe along with the tones so that they are inhaling with the high tone and exhaling with the low tone. They were also told to try and maintain a natural breath depth. The NIB group was given no explicit instructions regarding breath.

The changes in tone cycle duration were not mentioned in the instructions and were intended to be subtle, but not necessarily sub‐perceptual to the participants. With this approach we intended that the change in tone cycle duration would not become a salient aspect of the task, which could have distracted from the primary clicking and breathing tasks. Additionally, participants may have altered their behavior in attunement with their expectations of how one should act under these changing dynamics. However, it was not of vital importance if participants did notice.

In Experiment 1, Blocks 4 and 5 were included to ensure that any difference in outcome measures is due to an effect of tone/breath cycle rate rather than ‘time on task.’ In Experiment 2, we sought to replicate findings from Experiment 1 and note any further effects from participants remaining longer in each tone cycle frequency and without the return to the original rate. We aimed for ~20 min of data collection in each experiment to allow time for the breath intervention to have attentional effects whilst not overexerting participants who are new to guided breathwork (beginner meditation and breathwork sessions are ~10–20 mins). And to attain sufficient behavioral and pupillary time course data to test for trends over time.

### 2.4 Questionnaire

A post‐task qualitative questionnaire was implemented primarily to get feedback on the PACE task and to assess the naivety of the NIB group to our study's focus on respiration. Questions were: (1) *Were you able to easily follow along with the tones using mouse clicks?* (2) *Did you notice anything change about the tones?* (3) *What did you think the point of this study was?* (4) *How much attention did you pay to your breath during this study?* (5) *How often do you pay attention to your breath in daily life?*. A simple analysis of the responses to these questions features in Appendix S1.

### 2.5 PACE Task Behavioral Analysis

Reaction time mean and variability: The predefined behavioral variables of interest to assess attentional stability from the PACE task are the (i) precision of click timing in relation to the tone transition time, reaction time mean (RTm), and the (ii) circular vector length of RTs (RTVL). Since the PACE task has cyclical stimuli, RTs were calculated as absolute phase angle deviations from 0 rad—the tone transition time. RTm was calculated for the blocks where tone length was constant at the frequencies of interest (Experiment 1, blocks 1, 3, and 5; Experiment 2, blocks 1 and 3). RTVL was represented by the vector length of RT phases—a circular statistic for variation in phase distribution (See 2. Methods; Respiration recording and analysis; for more details on vector lengths). We included trials where participants committed ‘inverted responses’ (see next paragraph) in the RT and RTVL analysis by re‐inverting the response phase angles.

#### 2.5.1 Response Errors

As an exploratory analysis, ‘inverted responses’ (IRs) were extracted as the number of times participants clicked the incorrect mouse button with respect to the tone transition type (left after the high tone, right after the low tone). IRs were detected as mouse clicks at phase deviations > π/2 from tone transitions and were thus more aligned to the opposite tone transition than the expected one. We additionally considered ‘Switches’–the count of instances when participants altered their response pattern, both correct to incorrect and incorrect to correct.

#### 2.5.2 Non‐Compliance to Task

We also considered the number of registered clicks compared to the number of expected clicks (Experiment 1–340 tone transitions, Experiment 2–350) to use as a measure of task compliance.

Values for these analyses were extracted from the csv file generated by PsychoPy and computed using MATLAB 2022b.

### 2.6 Respiration Recording and Auditory‐Respiratory Entrainment Analysis

Respiration was continuously measured using a SleepSense effort sensory respiratory belt (https://sleepsense.com/) placed approximately at the bottom of the sternum. The signal was recorded with a Biosemi amplifier and Actiview software (https://www.biosemi.com/), sampled at 256 Hz. In preprocessing, the signal was checked by eye for clear recordings and then low‐pass filtered using a zero‐phase digital Butterworth filter with a cutoff frequency of 0.6 Hz and a filter order of 4. Filtered signals were then linearly detrended.

To represent the strength of respiratory entrainment to the tone stimuli, the consistency of respiratory phases occurring at the time of tone transitions was calculated: For this, the respiration signal was Hilbert transformed [‘hilbert()’; MATLAB] and phase angles were extracted [‘angle()’; MATLAB] so each sample point lay between −π and +π radians and could be represented on a circle. The resultant vector length from plotting the respiratory phases at tone transition times [‘circ_*r*()’; MATLAB] represents the degree of entrainment and lies between 0 (arbitrary distribution) and 1 (distributed to a singular phase angle). These auditory‐respiratory vector lengths were analyzed separately for HL and LH since it is known that respiratory phases differentially entrain to certain task events (see 1. Introduction).

Additional measurements for entrainment are the mean phase angle that the resultant vector tends towards [‘circ_mean()’; MATLAB] and a test result from the Rayleigh test of non‐uniformity around a circle [‘circ_rtest()’; MATLAB]. Circular statistics functions were used from the CircStat Toolbox (Berens 2009). For more information on circular statistics, see Cremers and Klugkist (2018).

### 2.7 Pupil Recording and Analysis

Pupil diameter (PD) was continuously recorded using an Eyelink 1000 Plus camera and associated SR Research software, sampled at 1000 Hz. Calibration and validation of the pupil was performed once, prior to the task's commencement. To process PD data, periods of eye closure due to blinking or otherwise were detected using the Eyelink algorithm ±50 ms. Eye closure indices were attributed null values, and these gaps were interpolated linearly between neighboring non‐zero values [‘interp1()’; MATLAB]. Interpolation ratios were calculated as the number of interpolated values divided by the total number of values in the PD array. An arbitrary cut‐off threshold of 0.3 (30% interpolated values) was chosen as ground for rejecting a participant's pupil dataset to avoid interpolated data contributing significant noise. PD was then low‐pass filtered using a zero‐phase digital Butterworth filter with a cut‐off frequency of 4 Hz and filter order of 4 and linearly detrended.

The primary analyses on PD were the power of frequency components for each block, obtained via fast Fourier transform [‘fft()’; MATLAB], a spectrogram analysis to see how frequency power changed over a finer timescale, and the phase relationship with respiration (next section).

### 2.8 Respiratory‐Pupillary Phase Coupling Analysis

Phase locking values (PLVs) and the corresponding mean phase angle differences were calculated to assess phase‐phase coupling and phase offset, respectively, between respiration and PD. Following the aforementioned pre‐processing steps for each signal, they were both bandpass filtered with a Butterworth filter of order 2 in the range of 0.01–0.4 Hz. This was to encompass low‐frequency sigh behavior and the highest respiration rate recorded for any block. Filtered signals were transformed into phase angles, and the angular difference [‘angdiff()’; MATLAB] was taken between them at every sample point. The resultant vector length of plotting these angular differences represents the consistency of the phase difference between these signals over time, thus assessing 1:1 phase‐phase coupling strength. The mean of these angular differences provides a measure of phase offset. Mean PLV was calculated for the task overall, for each block, and as a moving average across the task with a window size of 10 s and a step of 2 s for a finer analysis of this relationship over time. To determine the significance of task PLVs vs. chance, the actual PLVs obtained were compared against the mean of surrogate PLVs +1.65 std. Surrogates were generated for each participant by randomly shifting the phase representation of their respiratory signal and calculating PLVs with PD 10,000 times. We additionally plotted grand average PD over the respiratory cycle for each block. This was performed by averaging PD within a moving phase window around the respiratory cycle, using a window of 5° and a step of 1°.

## 3 Results

### 3.1 Experiment 1

In Experiment 1, participants completed a version of the Paced Auditory Cue Entrainment (PACE) task where the tone cycle frequency was initially constant at 0.15 Hz, slowed to a constant 0.1 Hz, and then sped back up to 0.15 Hz (task schematic in Figure 1).

Appendix S1 includes an analysis of manipulations checks and validity of the novel PACE task. Overall it was deemed that the IB group followed the respiratory guide well, the NIB group showed little respiratory entrainment to the tones, and the task was generally well complied to.

### 3.2 Behavioral Attentional Stability

Exclusions from the full cohort were made due to incomplete data collection, non‐compliance to task, or low breath entrainment (IB) (Appendix S1). Following exclusions, IB *n* = 23, NIB *n* = 32.

### 3.3 RTm and RTVL

**FIGURE 2:**
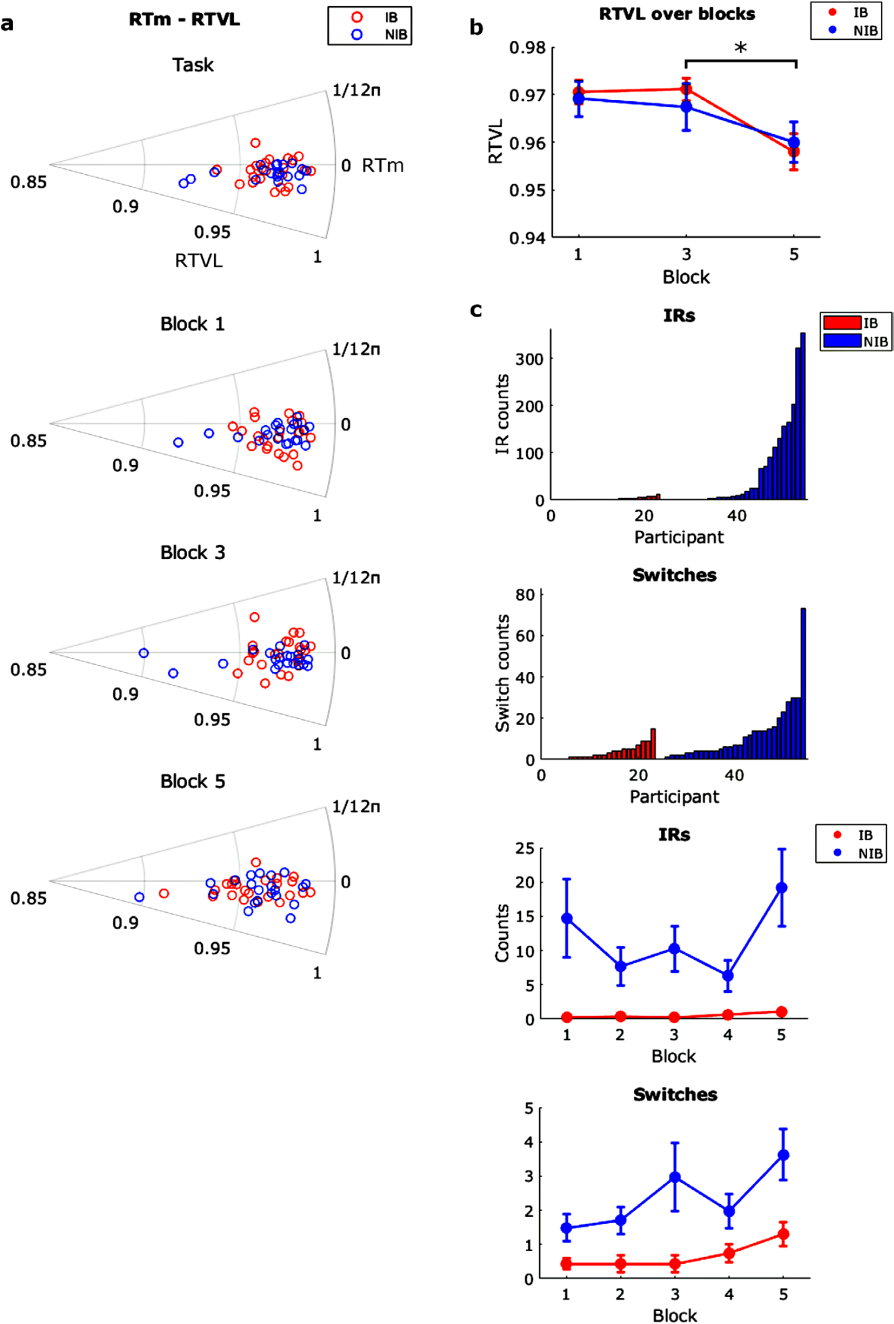
Exp. 1—(a) Polar scatter plots showing the reaction time mean (RTm; circular axis) and reaction time vector length (RTVL; radial axis), derived from mouse responses. (b) RTVL only over blocks 1, 3, and 5. Asterisk indicates a block difference at *p* < 0.05. Error bars indicate the standard error of the mean. (c) Top shows bar charts for the counts of Inverted Response (IR) and Switch errors committed by each participant. Bottom shows these error counts over blocks 1–5.

Polar scatter plots of RTm and RTVL for the whole task and each block are shown in Figure 2a.

For RTm at the task level, the IB group ranged 0.06–0.68 rad, *M* = 0.23 rad ±0.03, and the NIB group ranged 0.11–0.76 rad, *M* = 0.26 ± 0.02. For RTVL, the IB group ranged 0.93–99, *M* = 0.96 ± 0.003. NIB group ranged 0.89–0.99, *M* = 0.96 ± 0.005.

Testing for differences in RTm between groups across blocks, a two‐factor circular ANOVA, Harrison Kanji test was used. There was no significant effect of block, χ^2^(2) = 2.72, *p* = 0.07, η^2^_*p*_ = 0.035, nor group, χ^2^(1) = 1.30, *p* = 0.26, η^2^_*p*_ = 0.007, nor interaction χ^2^(2) = 0.03, *p* = 0.97, η^2^_*p*_ = 0. Therefore, there was no significant effect of tone cycle frequency or instructed breathing on response timing accuracy.

For RTVL, RM‐ANOVA showed a significant main effect of block, *F*(2) = 14.11, *p* < 0.001, η^2^_*p*_ = 0.213, with significant differences for block 1 (0.15 Hz) vs. block 5 (0.15 Hz) and block 3 (0.1 Hz) vs. block 5 (0.15 Hz), but no main effect of group, *F*(1) = 1.51, *p* = 0.23, η^2^_*p*_ = 0.028, nor interaction, *F*(2) = 2.54, *p* = 0.08, η^2^_*p*_ = 0.047. RTVL was lowest in block 5, which means the highest variability since it is a vector length representing RT clustering (Figure 2b). Therefore, there was a significant effect of tone cycle frequency but no significant effect of instructed breathing on response timing variability. This likely represents a time‐on‐task attention decrement for both groups.

These conclusions run counter to the hypothesis that slow‐paced breathing stabilizes behavior‐derived attention.

### 3.4 Inverted Responses (IRs) and Switches

IRs represent the number of incorrect responses with respect to the correct ‘high tone → left click, ‘low tone → right click’ rhythm. ‘Switches' represent the counts of when the participants' response pattern changed between correct and inverted. These were counted across all five blocks.

Instructed Breath IRs ranged 0–11 (0%–3.2% of expected clicks), *M* = 2.57 ± 0.59 (0.77% ± 0.17%). NIB IRs ranged 0–353 (0%–103.8%), *M* = 58.10 ± 16.88 (17.10% ± 4.96%). RM‐ANOVA showed a trend for a significant main effect of block *F*(2.34) = 2.78, *p* = 0.057, η^2^_*p*_ = 0.051, a significant effect of group *F*(1) = 7.99, *p* = 0.007, η^2^_*p*_ = 0.133, and no significant interaction *F*(2.34) = 2.45, *p* = 0.082, η^2^_*p*_ = 0.045. IRs seemed to steadily rise over the blocks for IB, whereas NIB showed the highest in blocks 1 and 5 and the lowest in 2 and 4 (Figure 2c).

Instructed Breath Switches ranged 0–20, *M* = 4.04 ± 1.02, and NIB ranged 0–63, *M* = 11.40 ± 2.30. RM‐ANOVA showed a significant main effect of block *F*(2.72) = 4.97, *p* = 0.004, η^2^_*p*_ = 0.087, and group *F*(1) = 7.42, *p* = 0.009, η^2^_*p*_ = 0.125, but no interaction *F*(2.72) = 1.61, *p* = 0.19, η^2^_*p*_ = 0.030. IB Switches were higher in blocks 4 and 5, whereas NIB Switches were higher in blocks 3 and 5 (Figure 2c).

Therefore, there was a significant effect of instructed breathing on these attentional lapses and some effect of tone cycle frequency.

There were significant negative correlations between IRs and auditory‐respiratory vector lengths in the IB group [LH: *r*(42) = −0.44, *p* = 0.03; HL, *r*(42) = −0.56, *p* = 0.005], as well as with Switches [HL *r*(42) = −0.53, *p* = 0.009]. This implies that the greater the auditory‐respiratory entrainment, the fewer lapses.

These conclusions support the hypothesis that slow‐paced breathing stabilizes behavior‐derived attention.

### 3.5 Pupil Diameter Oscillatory Frequency

#### 3.5.1 Pupil Diameter Power Spectra

**FIGURE 3:**
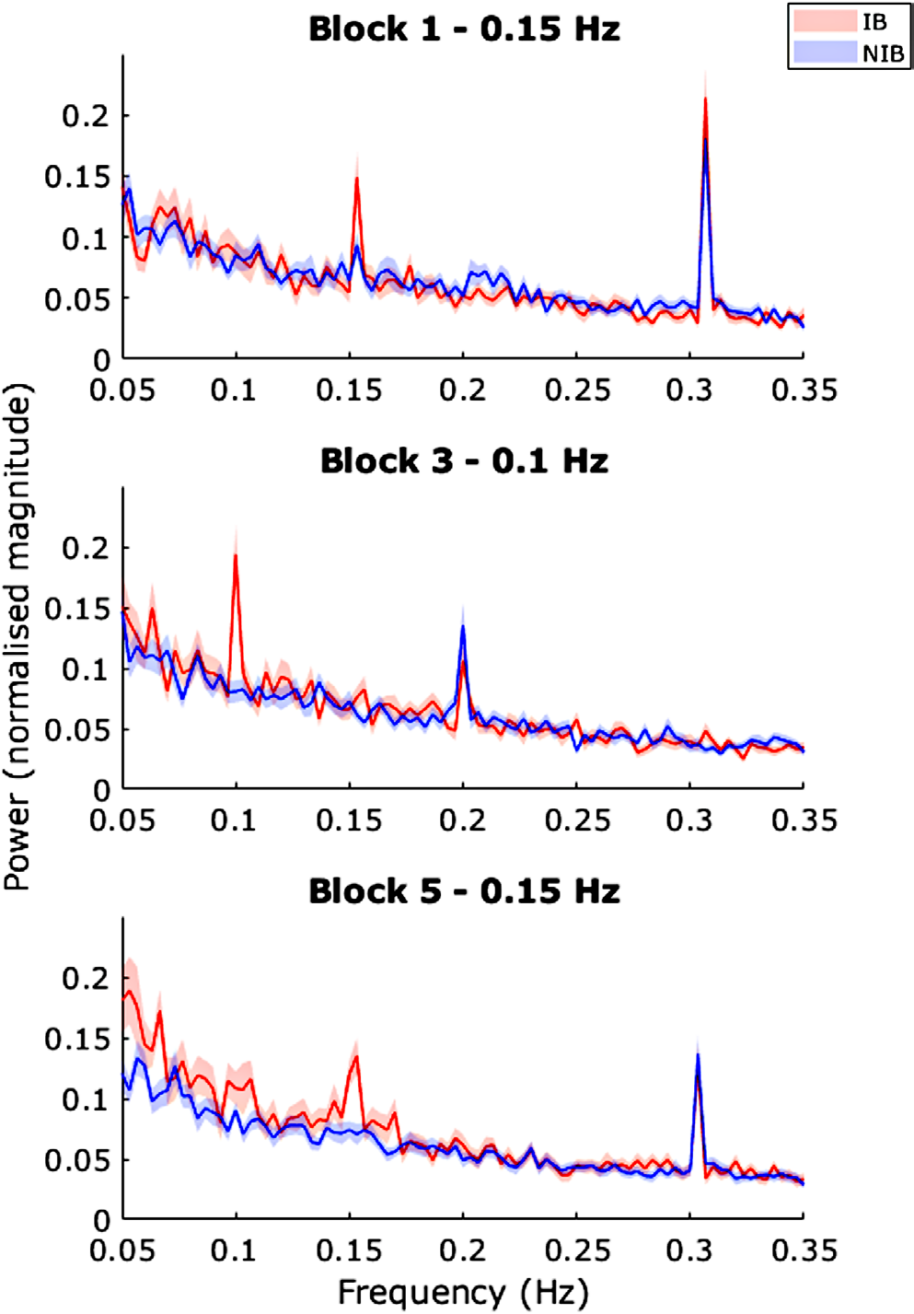
Exp. 1—Pupil diameter power spectra for block 1 (0.15 Hz), block 3 (0.1 Hz), and block 5 (0.1 Hz). Shaded areas represent SEM.

To investigate the presence of a respiratory frequency component in the PD oscillations, we considered the power of PD frequencies at the guided respiratory frequencies of interest, 0.1 and 0.15 Hz (Figure 3). For the subsequent analyses involving PD, four participants from the NIB group and four participants from the IB group were removed due to poor quality PD data (Following exclusions, IB *n* = 19, NIB *n* = 27).

*t*‐Tests at the peaks of interest showed significant differences: Block 1 0.15 Hz—*t*(44) = 2.51, *p* = 0.016, *d* = 0.75; Block 3 0.1 Hz—*t*(44) = 4.95, *p* < 0.001, *d* = 1.48; Block 5 0.15 Hz—*t*(44) = 3.94, *p* < 0.001, *d* = 1.18.

Also present in the spectra for both groups are peaks at 0.3 Hz in blocks 1 and 5 and 0.2 Hz in block 3 (Figure 3). Means comparison tests show no significant difference between the power of these peaks in each group at the task or block level (all *p* > 0.2). These shared peaks could possibly be induced by the auditory stimulus.

#### 3.5.2 Respiration and Pupil Diameter Spectrograms

**FIGURE 4:**
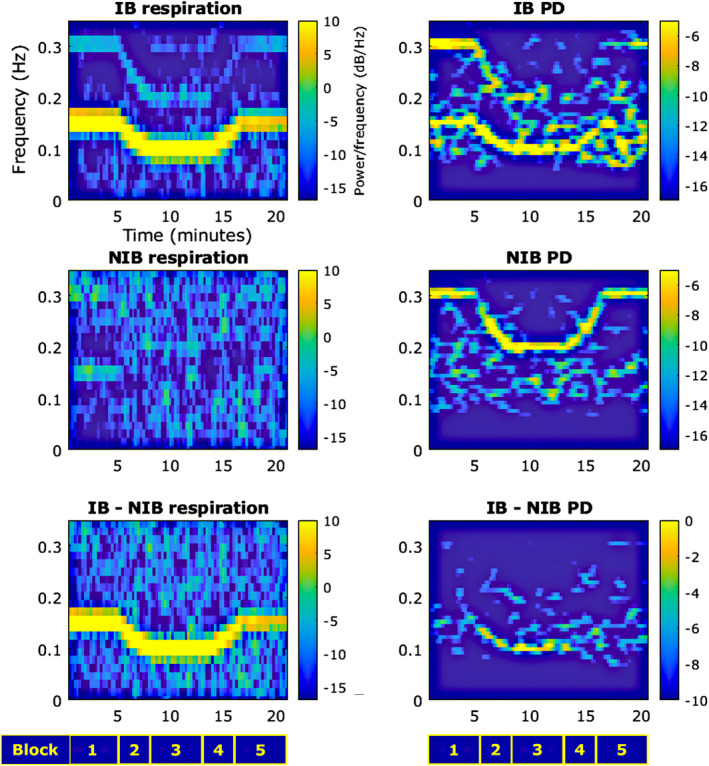
Exp. 1—Spectrograms across the whole task for respiration (left) and pupil diameter (PD) (right). The bottom row shows difference plots. PD signals were bandpass filtered between 0.075 and 0.4 Hz for display purposes.

To further evidence that the changing rate of respiration correlated to a shift in dominant PD frequencies within the same range, spectrograms for both were plotted across the task (Figure 4). For the IB group, the respiration plot follows the tone cycle closely; however, block 5 appears to have slightly reduced power at 0.15 Hz vs. block 1. The PD frequency power approximately follows respiration for IB. Block 1 is dominated by 0.15 Hz; in block 2, the 0.15 Hz component progressively decreases to 0.1 Hz; during block 3, there is a dominant 0.1 Hz component; and then the pattern is less clear during blocks 4 and 5, where there is significant power throughout the 0.1–0.15 Hz range in a variable manner. This shifting pattern is also evident in the 0.2–0.3 Hz range. The NIB group does not show a clear dominance of respiratory frequencies; however, there does appear to be some subtle but consistent activity at 0.15 Hz during block 1. The dominant shifts in PD for NIB are within the 0.2–0.3 Hz range but broadly show a similar pattern to the IB changes—starting at 0.3 Hz, shifting to 0.2 Hz, and back to 0.3 Hz. A spectrogram plotted of the difference in PD frequency power between the groups (IB minus NIB) shows increased power in the 0.1–0.15 Hz range, predominantly in blocks 2 and 3.

These patterns support the hypothesis that slow‐paced breathing is related to concomitant stable trends in PD oscillation.

### 3.6 Phase Coupling Relationship Between Respiration and Pupil Diameter

#### 3.6.1 Phase Locking Analysis

Since the guided respiratory frequencies overlap significantly with PD frequencies, we sought to characterize this coupling relationship with phase locking analysis.

**FIGURE 5:**
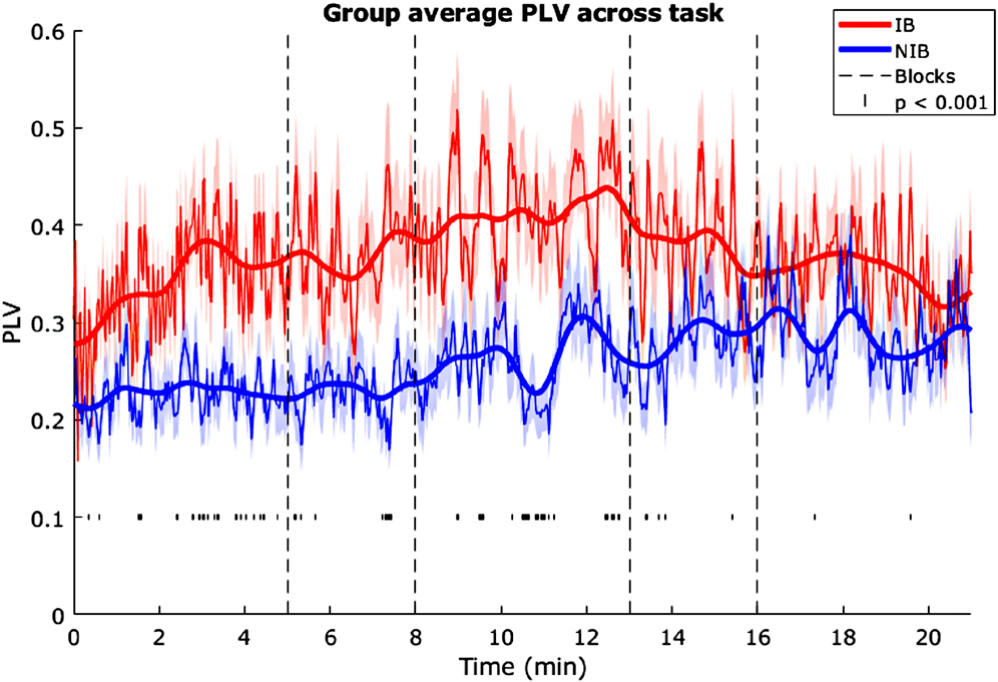
Exp. 1—Phase locking value (PLV) between respiration and pupil diameter, moving average across the task. Thinner lines and shaded areas indicate the means and SEM, respectively, the thicker line represents a smoothed mean for each group. Dotted vertical lines indicate blocks, and small dashes below indicate where groups differ at *p* < 0.001.

At the whole task level, phase locking values (PLVs) obtained were as follows the IB group ranged from 0.04 to 0.31, *M* = 0.13 ± 0.015, and the NIB 0.02–0.20, *M* = 0.07 ± 0.01. The IB group mean was significantly higher, *t*(44) = 4.24, *p* < 0.001, *d* = 1.27. IB group mean phase offset between respiration and PD was −0.30 rad ± 0.17, and NIB was −1.46 rad ± 0.14 (interpret as PD negatively shifted in phase with respect to respiration for both; Figure 5). These mean phase offsets were significantly different, *F*(44) = 21.67, *p* < 0.001, η^2^_*p*_ = 0.30, the IB phase angles being closer to 0 rad.

Investigating an effect of blocks on PLVs, RM‐ANOVA across blocks 1, 3, and 5 showed no significant main effect of blocks, *F*(2) = 0.28, *p* = 0.76, η^2^_*p*_ = 0.006, a significant main effect of group, *F*(1) = 22.58, *p* < 0.001, η^2^_*p*_ = 0.34, with higher PLVs in the IB group in all blocks, and a significant interaction, *F*(2) = 4.25, *p* = 0.02, η^2^_*p*_ = 0.088.

To determine whether task PLVs obtained were significantly above chance (1.65 std), actual PLVs were compared to surrogate PLVs. 16/19 IB group participants had PLVs that were significantly greater than the surrogate, with a group mean of 3.88 std. ± 0.43 above the surrogate mean. 19/27 NIB group participants' PLVs were significantly greater than the surrogate, *M* = 2.73 std. ± 0.38. The group difference in ‘stds above the surrogate’ was borderline significant, *t*(44) = 1.99, *p* = 0.05, *d* = 0.60, with the IB group mean being slightly higher.

A grand moving average plot of PLVs across the whole task (Figure 5) shows that the PLVs for the IB group remain higher than the NIB group until the very end. Further, there is an indication in the IB group of PLV increasing through blocks 1, 2, and 3, and then decreasing during blocks 4 and 5. A running *t*‐test across time shows most significant differences between the groups at *p* < 0.001 lie towards the second half of block 1, towards the end of block 2, and throughout block 3. PLV in the NIB group stays fairly consistently low and then starts gradually rising from the beginning of block 3, and by the end of the task it is comparable to that of IB.

**FIGURE 6:**
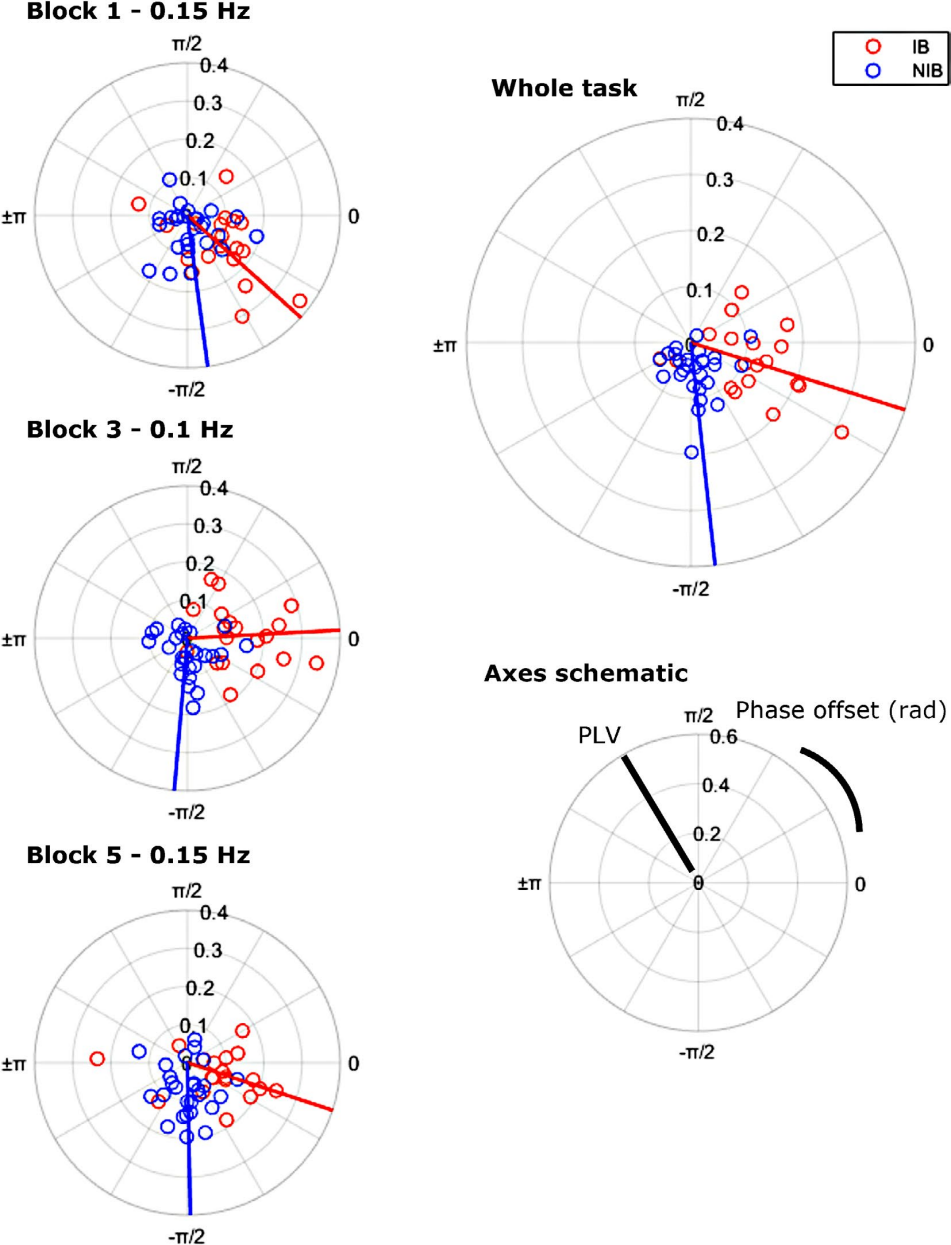
Exp. 1—Polar scatter showing the phase locking value (PLV) (radial axis) and phase offset (angular axis) between phasetransformed respiration and pupil diameter signals. Shown for each block (left) and the whole task (top right). Lines indicate mean phase offsets for each group. Negative radians indicate that pupil diameter is negatively shifted with respect to respiration.

The mean phase offset appeared to shift over the blocks (Figure 6); however, a Harrison Kanji test only showed a significant main effect of group χ^2^(1) = 38.17, *p* < 0.001, and no effect of block, χ^2^(2) = 3.03, *p* = 0.22, nor interaction, χ^2^(2) = 6.26, *p* = 0.98.

These conclusions support the hypothesis that slow‐paced breathing alters the strength and phase offset of a respiratory‐pupillary coupling relationship.

### 3.7 Pupil Diameter Over the Respiratory Cycle

**FIGURE 7:**
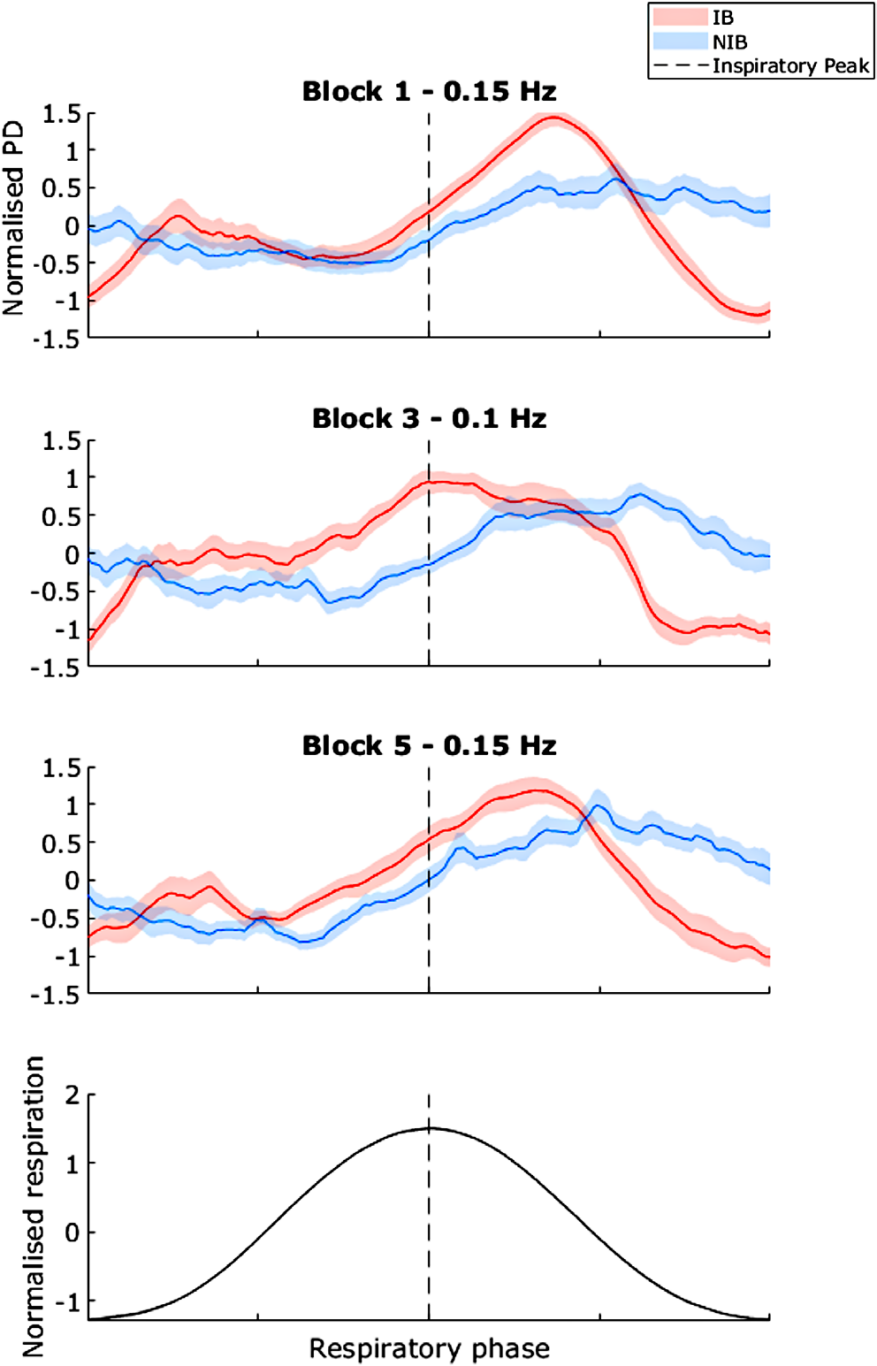
Exp. 1—Normalized pupil diameter (PD) over the respiratory cycle for each block. Shaded areas indicate SEM. The central dotted line represents the inspiratory peak. Respiration cycle plotted for reference (bottom) Page 28 of 69 Ps.

To characterize the respiratory‐PD coupling on a breath‐by‐breath basis, we plotted changes in PD over the respiratory cycle for each of the three blocks (Figure 7). In the IB group, PD shows an initial sharp rise with inhalation onset, a slight plateau, a gradual second rise from late inhalation to early exhalation, and then a steady decrease. The NIB group plot line shows a mild sinusoidal PD modulation, being lower during inhalation and higher in exhalation. Importantly, the PD modulation patterns are phase‐shifted relative to the respiratory cycle, and thus ruling out the possibility that changes in PD are simply due to movement artifacts over the respiratory cycle. As with the prior analysis, the phase offset appears to be closer to 0 during block 3.

These patterns support the hypothesis that slow‐paced breathing is related to concomitant stable trends in PD oscillation.

### 3.8 Experiment 2

In Experiment 2, a separate pool of participants completed a version of the PACE task where the tone cycle frequency was initially constant at 0.15 Hz, then slowed to a constant of 0.1 Hz, with no further frequency changes (task schematic in Figure 1).

Appendix S1 includes an analysis of manipulations checks and validity of the novel PACE task. Overall it was deemed that the IB group followed the respiratory guide well, the NIB group showed little respiratory entrainment to the tones, and the task was generally well complied to.

### 3.9 Behavioral Attentional Stability

Exclusions from the full cohort were made due to incomplete data collection, non‐compliance to task, low breath entrainment (IB), or high breath entrainment (NIB) (Appendix S1). Following exclusions, IB *n* = 23, NIB *n* = 25.

### 3.10 RTm and RTVL

**FIGURE 8:**
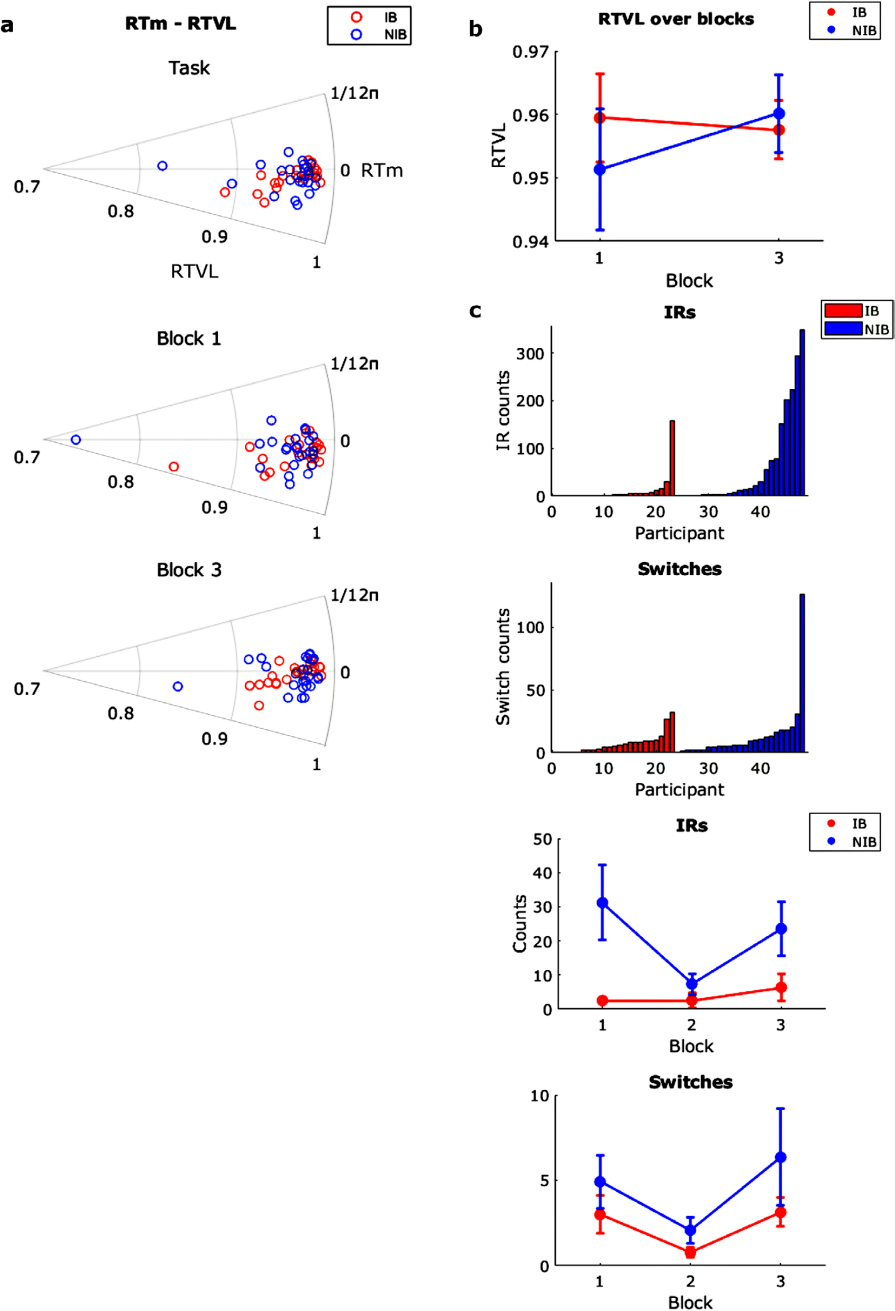
Exp. 2—(a) Polar scatter plots showing the reaction time mean (RTm; circular axis) and reaction time vector length (RTVL; radial axis), derived from mouse responses. (b) RTVL only over blocks 1 and 3. Asterisk indicates a block difference at *p* < 0.05. Error bars indicate the standard error of the mean. (c) Top shows bar charts for the counts of Inverted Response (IR) and Switch errors committed by each participant. Bottom shows these error counts over blocks 1–3.

Polar scatter plots of RTm and RTVL for the whole task and each block are shown in Figure 8a.

For RTm at the task level, the IB group ranged 0.08–0.75 rad, *M* = 0.29 rad ± 0.03, and the NIB group ranged 0.03–0.88 rad, *M* = 0.29 ± 0.04. For RTVL, the IB group ranged 0.89–0.99, *M* = 0.96 ± 0.005. NIB group ranged 0.82–0.98, *M* = 0.96 ± 0.007.

For block analyses, blocks 1 (0.15 Hz) and 3 (0.1 Hz) were compared. Testing for differences in RTm between groups across blocks, a two‐factor circular ANOVA, Harrison Kanji test was used. There was no significant effect of block, χ^2^(1) = 0.35, *p* = 0.55, η^2^_*p*_ = 0.003, or group, χ^2^(1) = 0.05, *p* = 0.82, η^2^_*p*_ = 0, or interaction χ^2^(1) = 0.02, *p* = 0.90, η^2^_*p*_ = 0. Therefore, there was no significant effect of tone cycle frequency or instructed breathing on response timing accuracy.

For RTVL, RM‐ANOVA showed no significant main effect of block, *F*(1) = 0.41, *p* = 0.53, η^2^_*p*_ = 0.009; no main effect of group, *F*(1) = 0.11, *p* = 0.75, η^2^_*p*_ = 0.002; and no interaction, *F*(1) = 0.98, *p* = 0.33, η^2^_*p*_ = 0.021 (Figure 8b). Therefore, there was no significant effect of tone cycle frequency or instructed breathing on response timing variability.

These conclusions run counter to the hypothesis that slow‐paced breathing stabilizes behavior‐derived attention.

### 3.11 Inverted Responses (IRs) and Switches

Instructed Breath IRs ranged 0–158 (0%–45% of expected clicks), *M* = 11.52 ± 6.81 (3.3% ± 2.0%). NIB IRs ranged 0–348 (0%–99.4%), *M* = 62.20 ± 20.07 (17.8% ± 5.7%). RM‐ANOVA showed a significant main effect of block *F*(1.58) = 4.69, *p* = 0.018, η^2^_*p*_ = 0.093, group *F*(1) = 5.34, *p* = 0.025, η^2^_*p*_ = 0.104, and interaction *F*(1.58) = 4.06, *p* = 0.03, η^2^_*p*_ = 0.081. IRs seemed to steadily rise over the blocks for IB, whereas NIB showed the highest in blocks 1 and 3 and the lowest in 2 (Figure 8c).

Instructed Breath Switches ranged 0–32, *M* = 6.91 ± 1.69; NIB ranged 0–126, *M* = 13.36 ± 4.92. RM‐ANOVA showed a significant main effect of block *F*(1.68) = 5.57, *p* = 0.008, η^2^_*p*_ = 0.108, but none for group *F*(1) = 1.44, *p* = 0.24, η^2^_*p*_ = 0.03 nor interaction *F*(1.68) = 0.45, *p* = 0.60, η^2^_*p*_ = 0.01. Switches were lower in block 2 compared to 1 (Holm *p* = 0.03, *d* = 0.34) and 3 (Holm *p* = 0.006, *d* = −0.44; Figure 8c).

Therefore, there was a significant effect of instructed breathing on the IR count and a significant effect of tone cycle frequency on both IRs and Switches.

There were significant negative correlations between IRs and auditory‐respiratory vector lengths in the IB group [LH: *r*(21) = −0.61, *p* = 0.002; HL: *r*(21) = −0.42, *p* = 0.046], as well as Switches with HL [*r*(21) = −0.48, *p* = 0.019]. This implies that the greater the auditory‐respiratory entrainment, the fewer lapses. No such significant correlations were present in the NIB group.

These conclusions support the hypothesis that slow‐paced breathing stabilizes behavior‐derived attention.

### 3.12 Pupil Diameter Oscillatory Frequency

#### 3.12.1 Pupil Diameter Power Spectra

For the subsequent analyses involving PD, two participants from the IB group and three from the NIB group were removed due to poor quality PD data (Following exclusions, IB *n* = 21, NIB *n* = 22).

**FIGURE 9:**
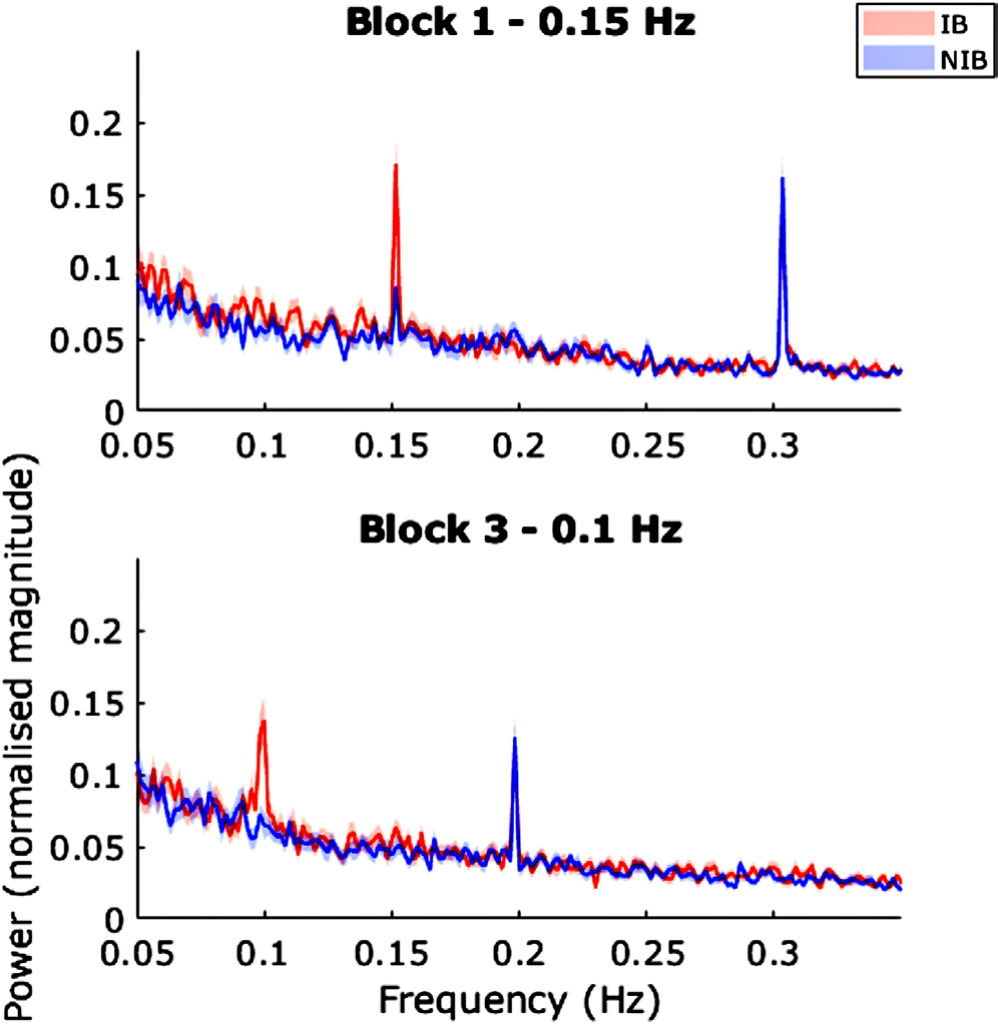
Exp. 2—Pupil diameter power spectra for block 1 (0.15 Hz) and block 3 (0.1 Hz). Shaded areas represent SEM. Note, the red and blue peaks at 0.2 (top) and 0.3 (bottom) Hz are at precisely the same location.

We looked at the PD power spectra for blocks 1 (0.15 Hz) and 3 (0.1 Hz) to investigate whether peaks are related to the respiratory frequency of that block (Figure 9).

*t*‐Tests at the peaks of interest showed significant differences: block 1, 0.15 Hz—*t*(41) = 2.84, *p* = 0.007. *d* = 0.87; block 3, 0.1 Hz—*t*(41) = 4.70, *p* < 0.001, *d* = 1.44.

Also present in the spectra for both groups are peaks at 0.3 Hz in block 1 and 0.2 Hz in block 3. Means comparison tests show no significant difference in the power of these peaks between each group: block 1, 0.3 Hz—*t*(41) = −0.004, *p* = 1.00, *d* = 0; block 3, 0.2 Hz—*t*(41) = 0.49, *p* = 0.63, *d* = 0.15.

#### 3.12.2 Respiration and Pupil Diameter Spectrograms

**FIGURE 10:**
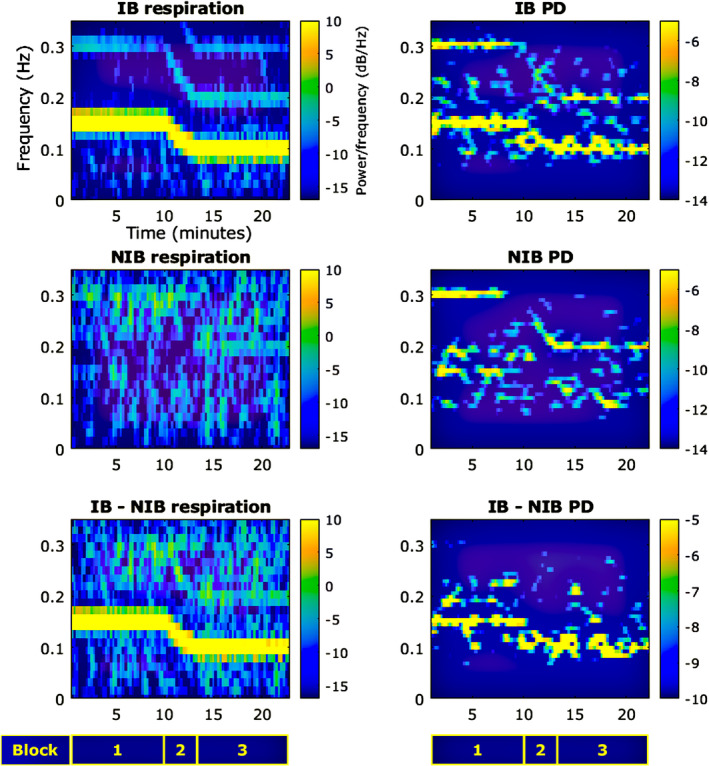
Exp. 2—Spectrograms across the whole task for respiration (left) and pupil diameter (PD) (right). The bottom row shows difference plots. PD signals were bandpass filtered between 0.075 and 0.4 Hz for display purposes.

Respiratory and pupillary spectrograms are shown in Figure 10. For the IB group, the respiration plot follows the tone cycle closely. The PD frequency power approximately follows respiration for IB, and then this pattern is partially mirrored in the 0.2–0.3 Hz range.

The NIB group does not show a clear dominance of respiratory frequencies; however, there does appear to be some consistent activity at 0.3 Hz during block 1 and at 0.2 Hz during block 3. The dominant shifts in PD for NIB are within the 0.2–0.3 Hz range but broadly show a similar pattern to the IB changes—starting at 0.3 Hz, shifting to 0.2 Hz, and back to 0.3 Hz.

A spectrogram plotted of the difference in PD frequency power between the groups (IB minus NIB) shows increased power in the 0.1–0.15 Hz range across the whole task.

These patterns support the hypothesis that slow‐paced breathing is related to concomitant stable trends in PD oscillation.

### 3.13 Phase Coupling Relationship Between Respiration and Pupil Diameter

#### 3.13.1 Phase Locking Analysis

At the whole task level, phase locking values (PLVs) obtained were: the IB group ranged 0.03–0.29, *M* = 0.12 ± 0.014, NIB 0.02–0.15, *M* = 0.07 ± 0.007.

Investigating an effect of blocks on PLVs, RM‐ANOVA across blocks 1, 2, and 3 showed no significant main effect of blocks, *F*(2) = 1.69, *p* = 0.19, η^2^_*p*_ = 0.04, and a significant main effect of group, *F*(1) = 21.06, *p* < 0.001, η^2^_*p*_ = 0.34, with higher PLVs in the IB group in all blocks and no interaction, *F*(1) = 1.14, *p* = 0.33, η^2^_*p*_ = 0.027.

To determine whether task PLVs obtained were significantly above chance (1.65 std), actual PLVs were compared to surrogate PLVs. 15/21 IB group participants had PLVs that were significantly greater than the surrogate, with a group mean of 2.48 std. ± 0.35 above the surrogate mean. 16/22 NIB group participants' PLVs were significantly greater than the surrogate, *M* = 3.48 std. ± 0.50. The group difference in ‘standard deviations above the surrogate’ was not significant, *t*(41) = −1.64, *p* = 0.11, *d* = −0.50.

**FIGURE 11:**
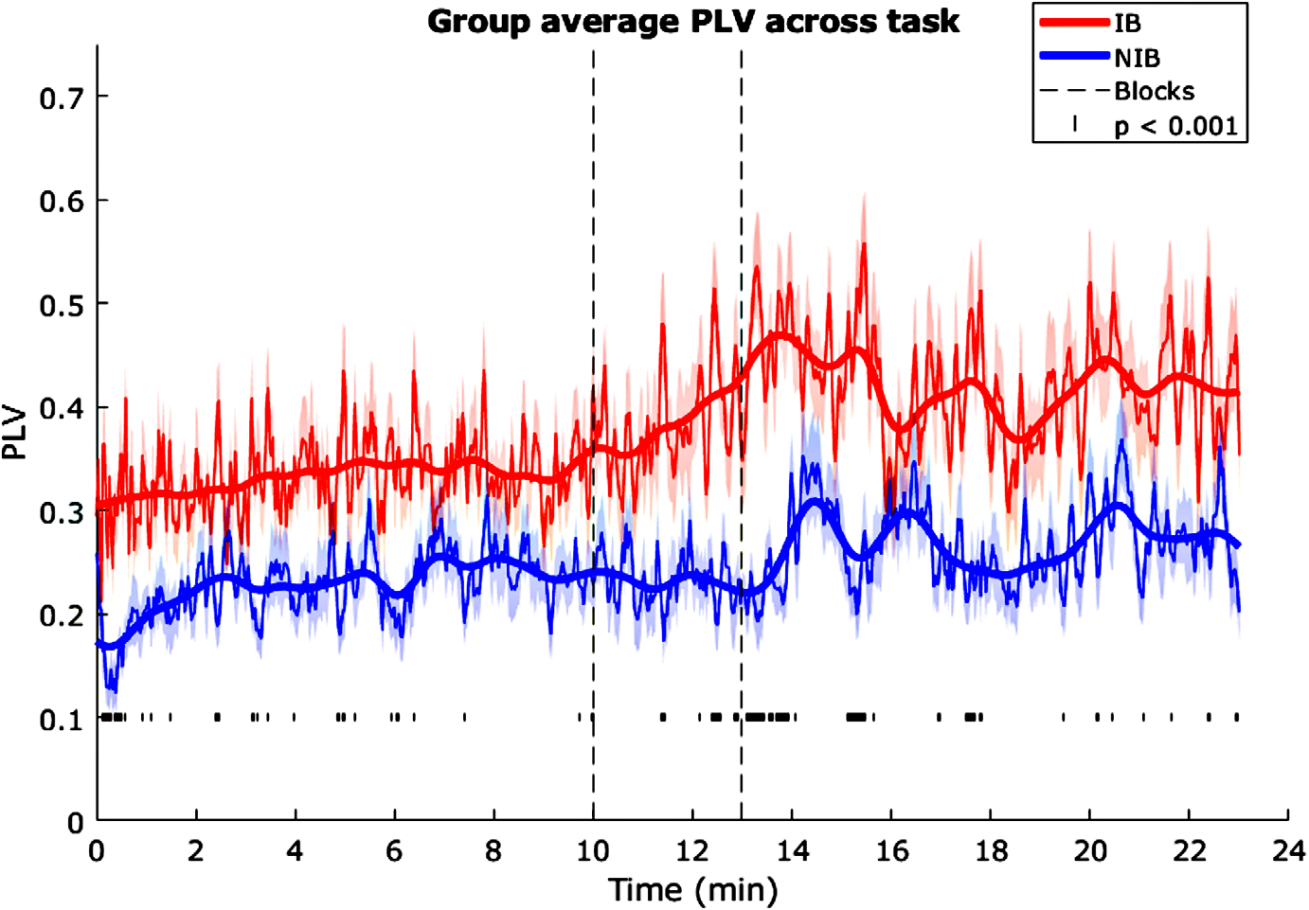
Exp. 2—Phase locking value (PLV) between respiration and pupil diameter, moving average across the task. Thinner lines and shaded areas indicate the means and SEM respectively, the thicker line represents a smoothed mean for each group. Dotted vertical lines indicate blocks and small dashes below indicate where groups differ at *p* < 0.001.

A grand moving average plot of PLVs (Figure 11) across the whole task shows that the PLVs for the IB group remain higher than the NIB group. Further, there is an indication in the IB group of PLV increasing through block 2 and staying higher during block 3, though with more fluctuation. A running *t*‐test across time shows most significant differences between the groups at *p* < 0.001 lie towards the end of block 2 and the beginning of 3. The NIB group also shows an apparent rise in PLV at the beginning of block 3, with the value then slightly fluctuating over time.

In Experiment 2, prior to commencement of the PACE task, participants had both respiration and PD recorded at resting for 3 mins (*n* = 63). Mean PLV during rest was not significantly different between the groups, *t*(56) = 1.05, *p* = 0.30, *d* = 0.28. When comparing PLVs during resting to surrogate values, 19/58 (using all participants) participants had PLVs significantly greater than the surrogate PLV. Interestingly, there was a highly significant negative correlation between their resting respiration rate and resting PLV, *r*(56) = −0.51, *p* < 0.001 (groups combined), as well as the number of standard deviations above the surrogate PLV mean, *r*(56) = −0.31, *p* = 0.02.

**FIGURE 12:**
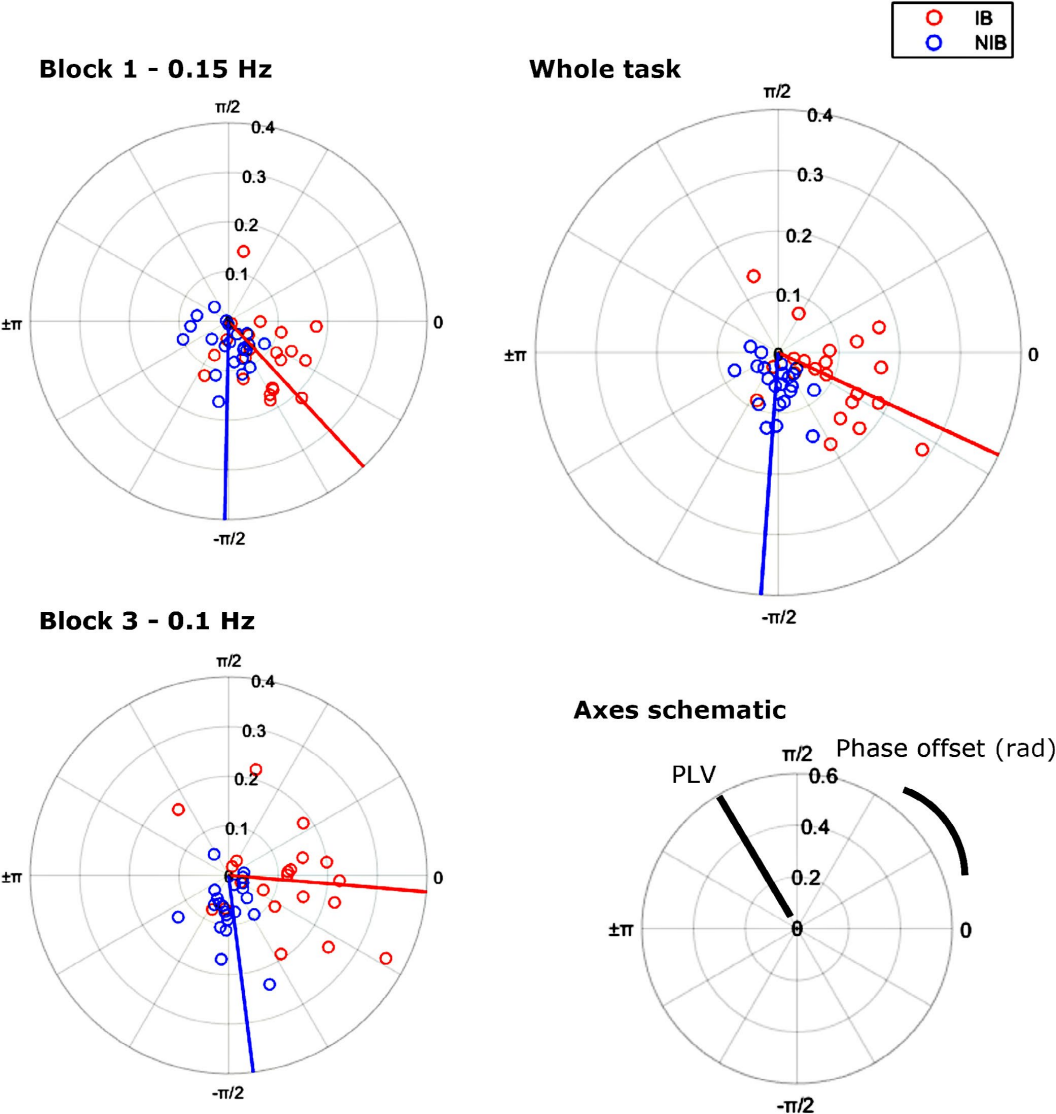
Exp. 2—Polar scatter showing the Phase Locking Value (PLV) (radial axis) and phase offset (angular axis) between phase‐transformed respiration and pupil diameter signals. Shown for each block (left) and the whole task (top right). Lines indicate mean phase offsets for each group. Negative radians indicate that pupil diameter is negatively shifted with respect to respiration.

During the PACE task, the IB group mean phase angle offset between respiration and PD was −0.44 rad ± 0.16, and for NIB it was −1.64 rad ± 0.12 (interpreted as PD negatively shifted in phase with respect to respiration for both). A Harrison Kanji (circular RM‐ANOVA) test of differences in phase offset showed a significant main effect of group χ^2^(1) = 28.81, *p* < 0.001, with the IB mean being closer to 0 rad, but no effect of block, χ^2^(1) = 3.98, *p* = 0.14, nor interaction, χ^2^(1) = 5.86, *p* = 0.99 (Figure 12).

These conclusions support the hypothesis that slow‐paced breathing alters the strength and phase offset of a respiratory‐pupillary coupling relationship.

#### 3.13.2 Pupil Diameter Over the Respiratory Cycle

**FIGURE 13:**
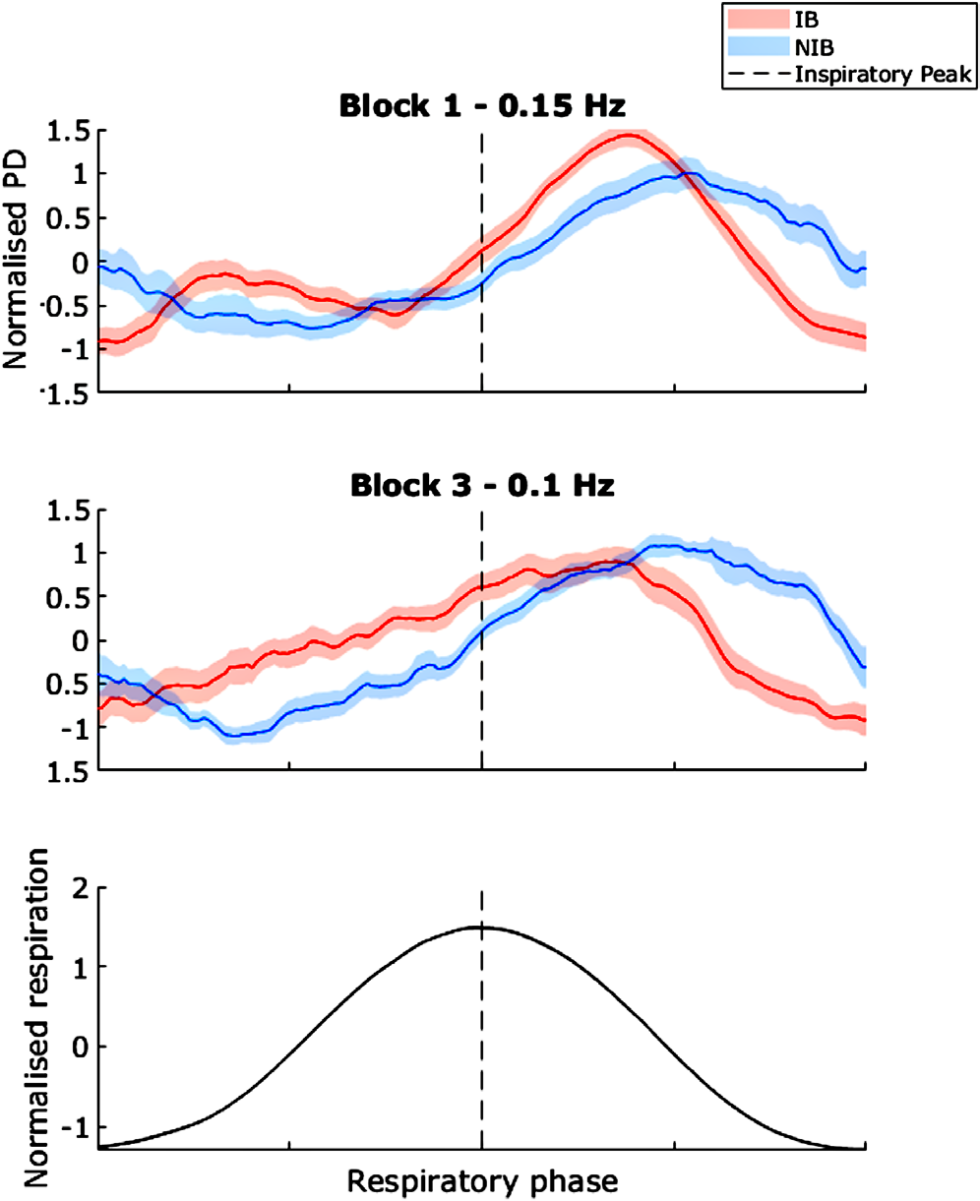
Exp. 2—Normalized pupil diameter (PD) over the respiratory cycle for each block. Shaded areas indicate SEM. The central dotted line represents the inspiratory peak. Respiration cycle plotted for reference (bottom).

See Figure 13. In block 1, for the IB group, PD shows an initial sharp rise with inhalation onset, a slight plateau, a second rise from late inhalation to early exhalation, and then a steady decrease. The NIB group showed a similar pattern, where PD reached a trough during inhalation and peaked over exhalation. In block 3, the IB pattern shows a steady linear increase over inhalation and a decrease from early exhalation to the end of exhalation. The NIB group pattern is similar to that of block 1. Importantly, the PD modulation patterns are phase shifted‐relative to the respiratory cycle thus, ruling out the possibility that changes in PD are simply due to movement artifacts over the respiratory cycle.

These patterns support the hypothesis that slow‐paced breathing is related to concomitant stable trends in PD oscillation, though not considerably more so than spontaneous breathing.

## 4 Discussion

The relationship between attention and breath is of central importance in traditional forms of meditation practice, but how respiration and attention influence each other from a psychophysiological perspective is underexplored and requires a novel paradigmatic approach. The present investigation sought to assess a link between slow‐paced breathing (SPB) and stable attention. To test our hypotheses, we constructed a novel task, the Paced Auditory Cue Entrainment (PACE) task, to serve as a simultaneous breath guide and attention monitoring task. To our knowledge, this is the second study to implement a breath guide during a task (D'Agostini et al. 2022), and we also demonstrated the feasibility of doing so. Using pupil‐linked arousal as a locus coeruleus (LC) proxy measure, we sought to provide evidence for a dynamical systems model that posits that respiration, LC‐noradrenaline (NA), and attention are autonomous oscillators that either tend towards stable attractor inter‐dynamics, regularizing the attentional oscillations that accompany the breath, or evolve towards a decoupled state, increasing variability and instability of attention (Melnychuk et al. 2018, 2021).

We hypothesized that a manipulation of SPB would push the system towards increased attentional stability concomitant with a reduction in pupil diameter (PD) oscillatory frequency. We found evidence for both, consistent across two experiments. SPB was highly related to maintaining the correct response rhythm over time, despite no group differences in the timing accuracy or variability of the response rhythm. Additionally, PD oscillations were closely entrained to the slow rhythm of the breath in the SPB group, enhancing the phase‐phase coupling strength.

### 4.1 Attentional Stability

With regards to our predefined behavioral metrics of attentional stability, we found no evidence that the presence or rate of our guided breath intervention impacted the timing accuracy (RTm) or variability (RTVL) of the response rhythm in our task. This was unexpected as we thought that either the breath task would complement performance in the clicking task, in line with the coupling model, our favored hypothesis, or alternatively, that there would be a ‘dual task interference effect’ in the Instructed Breath (IB) group, where performance would decline in both the ‘breath task’ and ‘clicking task.’ The comparable performance within and between groups for RTm and RTVL indicates that there is perhaps a ceiling effect in the PACE task performance, i.e., its demands are not sufficiently difficult or complex enough for variability in performance to arise between individuals or experimental conditions.

However, an exploratory analysis on the data in Experiment 1 of incorrect response keys provided behavioral discrimination between the groups. Here, the IB group committed fewer Inverted Response (IR) and Switch errors than the No Instructed Breath (NIB) group, and in Experiment 2, we replicated the group difference in IRs only. The lack of a group difference in the Switches despite a difference in IRs in Experiment 2 could possibly reflect the IB group applying Switches to rectify a previous Switch that deviated from the correct response rhythm. However, it is not possible from the current datasets to determine the intention of a Switch. The total number of erroneous clicks, IRs, therefore serves as the reliable behavioral discriminator for those with and without an SPB manipulation in this study. The significant negative correlations within the IB group for the degree of respiratory‐tone entrainment and the number of IRs and switches provide further evidence that adhering to the breath guide aided attentional stability with respect to these measures.

The positive findings for IRs and switches but not for RTm and RTVL imply that the groups differed in their longer‐term attentional state fluctuations rather than shorter‐term trial‐to‐trial variability. This is consistent with a study that has shown a positive influence of SBP on ‘error monitoring’ (Hoffmann et al. 2019), and so, perhaps the effects of SBP are more related to mitigating lapses in attention through enhancing meta‐awareness of performance.

In the following discussion of our respiration‐PD findings, we offer a possible explanation as to how regulating the breath may modulate attention via the LC‐NA system.

### 4.2 Respiratory‐PD Coupling

Through multiple analyses, we show that SPB had a remarkably strong effect on modulating PD oscillatory frequency, bringing it into the same rate as respiration.

The moving average analysis of the phase locking value (PLV), a measure of phase‐phase coupling, provided insights into trends of respiratory‐pupillary coupling strength over time. The phase coupling appeared to increase for the IB group when the breath was slowing, whereas in the NIB group, it seemed to steadily increase as a function of time.

A fairly consistent finding across the two experiments is that the PLVs obtained during the task were significantly greater than chance for ~70% of participants, regardless of group—although this proportion was higher for the IB group in Experiment 1 (84%). This implies a considerable inter‐individual variability in respiratory‐pupillary phase coupling strength. At rest, only ~30% of participants had PLVs significantly greater than chance. It makes sense that phase coupling was stronger when pupil and/or respiration was entrained to a constant frequency as was seen during the task. For the NIB group, PD seemed to entrain to the auditory tones in the task, exhibiting pupil oscillations at the frequency of tone presentation (0.2–0.3 Hz). However, the resting period was of much shorter duration (3 vs. 23 mins), which may limit comparisons to the task. A highly intriguing finding is that the participants' phase coupling strength at rest was strongly negatively correlated to their resting respiration rate. This is consistent with the increase in phase coupling seen in the IB group as the breath slows during the PACE task. It therefore seems that respiratory‐pupillary phase coupling strength is considerably dependent on respiratory rate. It would be curious to explore further how much this can explain inter‐individual variability and how the coupling relationship evolves alongside a habitual respiratory rate.

Additional insights were gained into the characteristics of a respiratory‐pupillary phase coupling relationship. In every task block, the IB group showed a clear modulation of PD over the course of the average respiratory cycle. The upward trajectory of PD was extended over the whole inhalation, into early exhalation, and appeared to be comprised of a sharp initial rise followed by a second gradual rise. Over mid‐late exhalation there was then a relatively rapid decrease. This pattern is highly consistent with a recent report of a ‘Respiratory‐Pupillary Phase Effect’ (Schaefer et al. 2024). A speculative interpretation is that the active process of inhalation causes an initial and then sustained drive to increase PD, which is then released during exhalation, the typically passive respiratory phase. A further interesting insight from this analysis was the observation that the NIB group also showed a respiratory‐dependent PD modulation pattern. Here, PD showed a somewhat smooth sinusoidal modulation, without any sharp increase, the amplitude of which appeared to slightly increase over the blocks. Therefore, respiratory modulation of PD appears to occur in the absence of a breathing manipulation (although some entrainment was evident for the NIB group here) given sufficient time, and possibly in monotonous settings such as the present one. The NIB group predominantly showed PD modulation in the 0.2–0.3 Hz range, which closely tracked the evolution of tone presentation frequency. We propose that the auditory tone entrained PD here, as the LC‐NA arousal and attentional system was exogenously captured with these stimuli.

Finally, an analysis of the phase offset, the phase difference between respiration and PD, showed that it was considerably closer to 0 in the IB group. Therefore, SPB is not only related to PD oscillations of similar frequency but also oscillations closer in phase. Phase offset was further smaller during the slowest breathing in the IB group; however, despite the consistency of this finding across the two experiments, there was no statistically significant difference here. Future study could widen the range of respiratory frequencies to see if a larger difference occurs.

Our findings are at odds with the conclusion from a recent review on the topic of respiratory dynamic modulation of PD, which concluded that evidence for an effect of respiration rate was “very low” (Schaefer et al. 2023). They included eight studies that directly tested this, and the results were mixed. Four studies showed an effect (Calcagnini et al. 2000; Daum and Fry 1981; Murata and Iwase 2000; Yoshida et al. 1994), and the other four, with the larger sample size (*n* > 15), were reported to have shown no effect (Bouma and Baghuis 1971; Schumann, Kralisch, and Bär 2015; Schumann, Andrack, and Bär 2017; Schumann et al. 2020), although it appears to us that Schumann et al. (2020) did show modulation through a slow breathing test (6 breaths per minute). Confusion likely arose due to the authors using “deep” and “slow” interchangeably—the review recognized an effect of depth for this study. All but one of these studies primarily correlated pupil activity and respiration at rest without any respiration intervention (except Schumann et al. 2020 also). This study from Daum and Fry (1981) was similar to the present study, using auditory stimuli to guide the breath in the range, 0.1–1 Hz and measuring pupil components. In contrast, they only included three participants, trials were 30 s long, and the pupil activity was broken down into discrete frequency components by their convolution method. Overall, their findings support a respiratory rate modulation of PD over a wide range of respiratory frequencies.

This review additionally did not include a study from Parnandi and Gutierrez‐Osuna (2013) who investigated estimating heart rate variability from PD. They had their five participants breathe at 6 (0.1 Hz), 9 (0.15 Hz), and 12 (0.2 Hz) breaths per minute, which resulted in clear PD spectral peaks at the respiratory frequencies. This also supports the present study's findings.

### 4.3 Respiration‐LC‐Attention Coupling

Beyond evidencing a respiratory‐pupillary coupling relationship, the present study sought to link this with attentional stability, in line with the respiration‐LC‐attention coupling model from Melnychuk et al. (2018). We investigated this from the perspective that PD is an established proxy measure for LC‐NA activity (Bang et al. 2023; DiNuzzo et al. 2019; Elman et al. 2017; Meissner et al. 2023; Murphy et al. 2014). This dynamical systems model posits that respiration, LC‐NA (pupil), and cortical attention networks are non‐linear, autonomous, noisy oscillators (see 1. Introduction). The oscillations are capable of interaction, falling in and out of synchrony, and producing emergent states of attention of variable stability. The specific hypothesis tested was that reducing the frequency and increasing the stability of respiratory pace stabilizes LC‐NA (pupillary) tonic oscillations and also reduces fluctuations in attention observable in behavior. We found evidence to support both aspects: PD oscillatory frequency stabilized at respiratory frequency, and attention‐sensitive errors were less frequent.

In the following, we entertain the possibility of a causal link between SPB and attentional stability via the LC through the perspective of the respiration‐LC‐attention coupling model (Melnychuk et al. 2018) and provide possible explanations of our findings.

The LC‐NA system is often discussed in relation to a proposed role as a facilitator of attentional flexibility. An influential model, the Adaptive Gain Theory (Aston‐Jones and Cohen 2005b), describes how the LC receives resultant information from areas such as the anterior cingulate cortex and orbitofrontal cortex, which aids an evaluation of the perceived reward/utility of exploiting a current focus. The LC either facilitates currently engaged networks through task‐locked phasic burst firing (phasic mode) or encourages exploration for an alternative focus through task‐indiscriminate tonic firing (tonic mode). A balanced strategy employing both phasic and tonic ‘modes’ likely underlies the experience of attentional state fluctuations and should aid the achievement of long‐term goals, utilizing cognitive flexibility. However, what if the long‐term goal is attentional *stability*, as it is here in the present study?

Previous studies investigating pupillometry signatures related to sustained attention have typically used sudden onset stimuli and noted the pre‐stimulus baseline (tonic) and stimulus‐evoked (phasic) components of PD (Benitez and Robison 2022; Martin, Whittaker, and Johnston 2022; Unsworth, Miller, and Aghel 2022; Unsworth and Robison 2016, 2018). From this perspective, sustained attention would be reflected in consistent PD signatures over time, i.e., constant tonic and phasic amplitudes, indicating a maintenance of the task‐focused phasic mode of the LC.

Instead, what we believe we show here is a cyclic fluctuation in tonic PD, entrained by the stimulus in both groups, but crucially, additionally entrained by respiration in the IB group, resulting in a higher attentional stability in this group. In the NIB group, attentional stability would have been largely due to top‐down control directed to the auditory stimuli, reflected in the auditory‐PD entrainment, and subject to ongoing motivational/utility evaluations. A drop in perceived utility could have led to a focal reorientation, and then depending on the time of task re‐engagement, participants may have returned with an incorrect response rhythm. In contrast, the IB group additionally could have had the bottom‐up influence of respiration on PD (LC) tonic levels. It should also be noted that since our participants were breathwork naive, there would have been a considerable top‐down attentional component on maintaining the respiratory rhythm also. These additional influences on LC‐NA may somewhat override endogenously driven attentional reorientations.

Such periodic fluctuations in tonic LC firing would induce a high degree of predictability into the system, reducing the chance of unexpected attentional orientations and therefore increasing attentional stability. Respiration has indeed recently been conceptualized as a signal that enhances predictability through multiple pathways, including the LC‐NA system (Brændholt et al. 2023) and higher‐order pathways to the insular cortex, which processes interoceptive signal information (Allen, Varga, and Heck 2023).

The pathways through which respiratory information could reach the LC are still speculative, though there are a number of possible candidates:

The preBötzinger Complex (preBötC), a medullary neural network and respiratory pattern generator (Muñoz‐Ortiz et al. 2019), projects to the LC in mice (Yackle et al. 2017). Ablation of this subset of neurons by Yackle et al. (2017) mediated arousal, inducing a prevailing calm state whilst leaving the breath rhythm intact. The preBötC burst fires in time with inspiration (Morgado‐Valle et al. 2015), and if these excitatory projections to the LC exist in humans, SPB may periodically innervate the LC, stabilizing arousal and attentional states.

A considerable number of LC neurons are CO_2_ sensitive in mice and rats, responding in a dose‐dependent manner to hypercapnia (Gargaglioni, Hartzler, and Putnam 2010) and relaying this information to other key respiratory areas (Krohn et al. 2023; Lopes et al. 2016). This raises the possibility that periodic fluctuations in CO_2_ over respiratory cycles could be registered by the LC in humans.

Vagal afferents from pulmonary stretch receptors relay respiratory information to the nucleus tractus solitarius (Kubin et al. 2006; Schelegle 2003), which has itself been suggested to project this to the LC (Melnychuk et al. 2018; Noble and Hochman 2019). During normal breathing, pulmonary vagal afferents are innervated via rapidly adapting receptors that produce phasic bursts in time with early inhalation, and during deep breathing, slowly adapting receptors are additionally activated throughout the duration of late inhalation. Our IB participants did indeed breathe significantly deeper than NIB (not reported here), and so they were likely to be recruiting both inspiratory‐associated receptors. This raises the possibility of cyclic vagal information reaching the LC. A two‐step increase could align with our PD pattern over the respiratory cycle, apparent in Experiment 1 and clearly evident in Experiment 2 at 0.15 Hz. However, precise connections to the LC are unclear here.

These aforementioned candidate pathways still remain speculative and primarily based on animal and in vitro models. However, our present findings in conjunction with Melnychuk et al.'s findings of respiratory‐PD (2018; 2021) and respiratory‐LC fMRI BOLD (2018) synchronization provide evidence of a linkage nonetheless. Hopefully, advancing imaging and analysis techniques in humans can elucidate the validity of these specific pathways.

## 5 Implications

Beyond consolidating the respiration‐LC‐attention model, the present conclusion that respiration can stabilize attention via the LC has important implications.

It challenges the notion that cognition is purely an intra‐brain process. Instead, an embodied cognition account is supported here, which instead proposes that cognitive processes and variability in behavior cannot be fully explained without consideration of peripheral signals. Here, we have investigated a potentially crucial input from respiration in this regard. If respiratory dynamics are indeed exerting continuous influence on arousal and attentional processes, investigations into behavioral or perceptual variability over time could benefit by simultaneously analyzing respiratory dynamics and noting explanatory power.

Slow‐paced breathing exercises are readily recommended for combatting stress and anxiety. However, there is very little understood about the underlying psychophysiological mechanisms. The LC pathway implies that slow‐paced breathing may also benefit individuals seeking to enhance attentional stability. It would be curious to research how respiratory modulation of LC activity may be affected by drugs such as noradrenaline reuptake inhibitors, which can be prescribed for attentional symptoms.

Meditation is a millennia‐old practice of cultivating attention. Focusing on the breath or modulating the breath is a central tool in meditative methods. This apparent feedback loop between attention and breath has largely avoided contemporary scientific investigation. The present suggests that the LC‐NA system is a candidate mechanism here. Noting how the coupled relationship between respiratory, LC‐NA, and attentional systems evolves over short‐ and long‐term meditation experiences would be highly informative (see Melnychuk et al. 2018 for a discussion here).

### 5.1 Strengths and Limitations of the Paradigm

It is worth critically assessing the efficacy of the novelly designed PACE task. As a breath guide, the IB participants generally entrained well by attuning their breath to the appropriate rate. However, entrainment did decrease steadily across the task. Other ‘time on task’ decrements in engagement were evident, with the latter blocks showing the lowest RTVL and the highest counts of IRs and switches for both groups.

Considering that we implemented guided breathwork to a group who were naive to such a practice, they maintained the rhythm well. It is worth noting that participants' resting respiration rate did not correlate with the degree of entrainment in the task.

IB participants entrained their breath stronger to low → high (LH) than high → low (HL) tone transition types. Respiratory‐LH entrainment meant aligning inhalation onset with the beginning of the tone, which would be consistent with the inhalation bias of the majority of recent findings regarding respiration‐task event entrainment (Kluger et al. 2021; Perl et al. 2019; Zelano et al. 2016).

Although IB attuned their respiration rate according to the task instructions, there was a consistent phase difference between respiration phase onset and tone transition whereby they began inhaling/exhaling prior to the tone transition time. Additionally, the breath onset was significantly earlier during the 0.1 Hz rate. This is not of major concern with regards to our study manipulation, manipulating respiratory rate; however, it would be if one is studying phase‐specific task events. It would be interesting to observe whether this anticipatory phenomenon occurs in individuals familiar with guided breathwork. It is worth noting that the PACE task imposed an equal inhalation—exhalation ratio which is atypical in spontaneous breathing, where the exhalation tends to be slightly longer. Perhaps this unfamiliar breathing rhythm can explain the phase difference here.

NIB participants did show some evidence of auditory‐respiratory entrainment, despite no explicit instructions to do so. Over half the participants in this group had significant non‐uniformity with regards to entraining their breath to the tone transitions. Additionally, on the NIB respiratory spectrograms, there are indications of consistent activity. The nature of our chosen stimuli was based on existing breath guides and was meant to be intuitive for the IB participants to follow, especially since they were breathwork naive. Those NIB participants who explicitly mentioned entrainment in the qualitative questionnaire or had relatively high auditory‐respiratory vector lengths were excluded from analyses. Otherwise we deemed the group's entrainment to be mild enough to consider them as a control group. This, of course, however, does not rule out the possibility of any effect from this level of entrainment. NIB auditory‐respiratory entrainment appeared to be biased towards late exhalation for both LH and HL tone transitions. Interpretations from this are limited due to the mildness of entrainment; however, it is worth noting that a previous study has found an exhale preference for response entrainment (Johannknecht and Kayser 2022). The mean entrainment angle for LH was significantly later in the breath cycle than HL, encroaching into early inhale. An area for future investigation may be how stimulus features affect respiratory entrainment behavior.

As an attention task, the PACE task appeared comprehensible to most participants judging from the subjective reports. However, there was the unintended but frequent issue of participants losing the correct left/right response rhythm with no prompt to aid a return to this. Fortunately, this ended up becoming a useful variable in discriminating the groups (IRs, Switches). Response rate was actually higher than 100% for most participants, implying that false alarms are more likely than omissions in this task. However, this only amounted to both groups averaging an extra 5% response rate (17 clicks) in Experiment 1, and less so in Experiment 2.

One notable limitation of the current study is the way the groups are controlled. The IB group essentially had two cues to support maintaining the correct response rhythm over time, the auditory rhythm and their respiratory rhythm. Should attention fail to recognize the auditory tones momentarily, the interoceptive cue of the breath may have acted as a backup. The question then arises as to whether this particular ‘attentional anchoring’ is a breath‐specific phenomenon. Up to a point, increasing the demand of a task should encourage engagement, especially demands geared towards the same rhythm. From the present experiment, it cannot be ruled out that if the NIB group had an additional, internal, temporal cue, such as numerical counting, they could show comparable response rhythm errors to the IB group. We believe that our PD findings corroborate the idea that attentional stability is related to a respiratory‐specific mechanism; however, future iterations using the PACE task should seek to more strictly control this group difference.

## 6 Concluding Remarks

Here we have demonstrated with a novel task that slow‐paced breathing was not associated with trial‐to‐trial response time accuracy but with a reduction in response key errors. We provided a possible mechanistic explanation for this by showing significant respiratory modulation of pupil diameter, implicating the locus coeruleus arousal and attentional system in mediating a psychophysiological effect. These findings contribute to a scarce literature on a topic that has been central to meditative practices for millennia and contribute to the emerging recognition of the role of respiration in shaping brain dynamics in cognitive neuroscience.

## Author Contributions

**Ralph Andrews:** conceptualization, data curation, formal analysis, investigation, methodology, project administration, visualization, writing – original draft, writing – review and editing. **Michael Melnychuk:** conceptualization, investigation, methodology, resources, software, supervision. **Sarah Moran:** data curation, investigation, project administration. **Teigan Walsh:** investigation. **Sophie Boylan:** investigation. **Paul Dockree:** conceptualization, funding acquisition, investigation, methodology, project administration, resources, supervision, validation, writing – review and editing.

## Conflicts of Interest

The authors declare no conflicts of interest.

## Supporting information


Appendix S1.

## Data Availability

The data that support the findings of this study are available from the corresponding author upon reasonable request.

## References

[psyp70003-bib-0063] Allen, M. , S. Varga , and D. H. Heck . 2023. “Respiratory Rhythms of the Predictive Mind.” Psychological Review 130, no. 4: 1066–1080. 10.1037/rev0000391. 35980689

[psyp70003-bib-0001] Aston‐Jones, G. , and J. D. Cohen . 2005a. “Adaptive Gain and the Role of the Locus Coeruleus‐Norepinephrine System in Optimal Performance.” Journal of Comparative Neurology 493, no. 1: 99–110. 10.1002/cne.20723. 16254995

[psyp70003-bib-0002] Aston‐Jones, G. , and J. D. Cohen . 2005b. “An Integrative Theory of Locus Coeruleus‐Norepinephrine Function: Adaptive Gain and Optimal Performance.” Annual Review of Neuroscience 28: 403–450. 10.1146/annurev.neuro.28.061604.135709. 16022602

[psyp70003-bib-0003] Bang, D. , Y. Luo , L. S. Barbosa , et al. 2023. “Noradrenaline Tracks Emotional Modulation of Attention in Human Amygdala.” Current Biology 33, no. 22: 5003–5010. 10.1016/j.cub.2023.09.074. 37875110 PMC10957395

[psyp70003-bib-0004] Benitez, V. L. , and M. K. Robison . 2022. “Pupillometry as a Window Into Young Children's Sustained Attention.” Journal of Intelligence 10, no. 4: 107. 10.3390/jintelligence10040107. 36412787 PMC9680391

[psyp70003-bib-0005] Berens, P. 2009. “CircStat: A MATLAB Toolbox for Circular Statistics.” Journal of Statistical Software 31: 1–21. 10.18637/jss.v031.i10.

[psyp70003-bib-0006] Bouma, H. , and L. C. J. Baghuis . 1971. “Hippus of the Pupil: Periods of Slow Oscillations of Unknown Origin.” Vision Research 11, no. 11: 1345–1351. 10.1016/0042-6989(71)90016-2. 5148578

[psyp70003-bib-0007] Boyadzhieva, A. , and E. Kayhan . 2021. “Keeping the Breath in Mind: Respiration, Neural Oscillations, and the Free Energy Principle.” Frontiers in Neuroscience 15: 647579. 10.3389/fnins.2021.647579. 34267621 PMC8275985

[psyp70003-bib-0008] Brændholt, M. , D. S. Kluger , S. Varga , D. H. Heck , J. Gross , and M. G. Allen . 2023. “Breathing in Waves: Understanding Respiratory‐Brain Coupling as a Gradient of Predictive Oscillations.” Neuroscience & Biobehavioral Reviews 152: 105262. 10.1016/j.neubiorev.2023.105262. 37271298

[psyp70003-bib-0009] Calcagnini, G. , F. Censi , S. Lino , and S. Cerutti . 2000. “Spontaneous Fluctuations of Human Pupil Reflect Central Autonomic Rhythms.” Methods of Information in Medicine 39, no. 2: 142–145. 10.1055/s-0038-1634277. 10892249

[psyp70003-bib-0010] Cazettes, F. , D. Reato , J. P. Morais , A. Renart , and Z. F. Mainen . 2021. “Phasic Activation of Dorsal Raphe Serotonergic Neurons Increases Pupil Size.” Current Biology 31, no. 1: 192–197. 10.1016/j.cub.2020.09.090. 33186549 PMC7808753

[psyp70003-bib-0011] Cremers, J. , and I. Klugkist . 2018. “One Direction? A Tutorial for Circular Data Analysis Using R With Examples in Cognitive Psychology.” Frontiers in Psychology 9: 2040. 10.3389/fpsyg.2018.02040. 30425670 PMC6218623

[psyp70003-bib-0012] D'Agostini, M. , N. Claes , M. Franssen , A. von Leupoldt , and I. Van Diest . 2022. “Learn to Breathe, Breathe to Learn? No Evidence for Effects of Slow Deep Breathing at a 0.1 Hz Frequency on Reversal Learning.” International Journal of Psychophysiology 174: 92–107. 10.1016/j.ijpsycho.2022.01.008. 35077759

[psyp70003-bib-0013] Daum, K. M. , and G. A. Fry . 1981. “The Component of Physiological Pupillary Unrest Correlated With Respiration.” Optometry and Vision Science 58, no. 10: 831–840. 10.1097/00006324-198110000-00008 7304710

[psyp70003-bib-0014] DiNuzzo, M. , D. Mascali , M. Moraschi , et al. 2019. “Brain Networks Underlying Eye's Pupil Dynamics.” Frontiers in Neuroscience 13: 965. 10.3389/fnins.2019.00965. 31619948 PMC6759985

[psyp70003-bib-0015] Elman, J. A. , M. S. Panizzon , D. J. Hagler , et al. 2017. “Task‐Evoked Pupil Dilation and BOLD Variance as Indicators of Locus Coeruleus Dysfunction.” Cortex 97: 60–69. 10.1016/j.cortex.2017.09.025. 29096196 PMC5716879

[psyp70003-bib-0016] Flexman, J. E. , R. G. Demaree , and D. D. Simpson . 1974. “Respiratory Phase and Visual Signal Detection.” Perception & Psychophysics 16, no. 2: 337–339. 10.3758/BF03203952.

[psyp70003-bib-0017] Fox, K. C. R. , R. N. Spreng , M. Ellamil , J. R. Andrews‐Hanna , and K. Christoff . 2015. “The Wandering Brain: Meta‐Analysis of Functional Neuroimaging Studies of Mind‐Wandering and Related Spontaneous Thought Processes.” NeuroImage 111: 611–621. 10.1016/j.neuroimage.2015.02.039. 25725466

[psyp70003-bib-0018] Fox, M. D. , A. Z. Snyder , J. L. Vincent , M. Corbetta , D. C. Van Essen , and M. E. Raichle . 2005. “The Human Brain Is Intrinsically Organized Into Dynamic, Anticorrelated Functional Networks.” Proceedings of the National Academy of Sciences 102, no. 27: 9673–9678. 10.1073/pnas.0504136102. PMC1157105 15976020

[psyp70003-bib-0019] Gargaglioni, L. H. , L. K. Hartzler , and R. W. Putnam . 2010. “The Locus Coeruleus and Central Chemosensitivity.” Respiratory Physiology & Neurobiology 173, no. 3: 264–273. 10.1016/j.resp.2010.04.024. 20435170 PMC2929404

[psyp70003-bib-0020] Goheen, J. , A. Wolman , L. L. Angeletti , A. Wolff , J. A. E. Anderson , and G. Northoff . 2024. “Dynamic Mechanisms That Couple the Brain and Breathing to the External Environment.” Communications Biology 7, no. 1: 1–11. 10.1038/s42003-024-06642-3. 39097670 PMC11297933

[psyp70003-bib-0021] Grund, M. , E. Al , M. Pabst , et al. 2022. “Respiration, Heartbeat, and Conscious Tactile Perception.” Journal of Neuroscience 42, no. 4: 643–656. 10.1523/JNEUROSCI.0592-21.2021. 34853084 PMC8805629

[psyp70003-bib-0022] Herrero, J. L. , S. Khuvis , E. Yeagle , M. Cerf , and A. D. Mehta . 2018. “Breathing Above the Brain Stem: Volitional Control and Attentional Modulation in Humans.” Journal of Neurophysiology 119, no. 1: 145–159. 10.1152/jn.00551.2017. 28954895 PMC5866472

[psyp70003-bib-0023] Hoffmann, S. , L. T. Jendreizik , U. Ettinger , and S. Laborde . 2019. “Keeping the Pace: The Effect of Slow‐Paced Breathing on Error Monitoring.” International Journal of Psychophysiology 146: 217–224. 10.1016/j.ijpsycho.2019.10.001. 31669325

[psyp70003-bib-0024] Jepma, M. , and S. Nieuwenhuis . 2011. “Pupil Diameter Predicts Changes in the Exploration‐Exploitation Trade‐Off: Evidence for the Adaptive Gain Theory.” Journal of Cognitive Neuroscience 23, no. 7: 1587–1596. 10.1162/jocn.2010.21548. 20666595

[psyp70003-bib-0025] Johannknecht, M. , and C. Kayser . 2022. “The Influence of the Respiratory Cycle on Reaction Times in Sensory‐Cognitive Paradigms.” Scientific Reports 12, no. 1: 8. 10.1038/s41598-022-06364-8. 35173204 PMC8850565

[psyp70003-bib-0026] Joshi, S. , Y. Li , R. M. Kalwani , and J. I. Gold . 2016. “Relationships Between Pupil Diameter and Neuronal Activity in the Locus Coeruleus, Colliculi, and Cingulate Cortex.” Neuron 89, no. 1: 221–234. 10.1016/j.neuron.2015.11.028. 26711118 PMC4707070

[psyp70003-bib-0027] Kluger, D. S. , E. Balestrieri , N. A. Busch , and J. Gross . 2021. “Respiration Aligns Perception With Neural Excitability.” 10: e70907. 10.7554/eLife.70907. PMC8763394 34904567

[psyp70003-bib-0028] Kluger, D. S. , and J. Gross . 2021. “Respiration Modulates Oscillatory Neural Network Activity at Rest.” PLoS Biology 19, no. 11: e3001457. 10.1371/journal.pbio.3001457. 34762645 PMC8610250

[psyp70003-bib-0029] Krohn, F. , M. Novello , R. S. van der Giessen , C. I. De Zeeuw , J. J. Pel , and L. W. Bosman . 2023. “The Integrated Brain Network That Controls Respiration.” eLife 12: e83654. 10.7554/eLife.83654. 36884287 PMC9995121

[psyp70003-bib-0030] Kubin, L. , G. F. Alheid , E. J. Zuperku , and D. R. McCrimmon . 2006. “Central Pathways of Pulmonary and Lower Airway Vagal Afferents.” Journal of Applied Physiology 101, no. 2: 618–627. 10.1152/japplphysiol.00252.2006. 16645192 PMC4503231

[psyp70003-bib-0031] Larsen, R. S. , and J. Waters . 2018. “Neuromodulatory Correlates of Pupil Dilation.” Frontiers in Neural Circuits 12: 21. 10.3389/fncir.2018.00021. 29593504 PMC5854659

[psyp70003-bib-0032] Lopes, L. T. , L. G. A. Patrone , K.‐Y. Li , et al. 2016. “Anatomical and Functional Connections Between the Locus Coeruleus and the Nucleus Tractus Solitarius in Neonatal Rats.” Neuroscience 324: 446–468. 10.1016/j.neuroscience.2016.03.036. 27001176 PMC4841468

[psyp70003-bib-0033] Martin, J. T. , A. H. Whittaker , and S. J. Johnston . 2022. “Pupillometry and the Vigilance Decrement: Task‐Evoked but Not Baseline Pupil Measures Reflect Declining Performance in Visual Vigilance Tasks.” European Journal of Neuroscience 55, no. 3: 778–799. 10.1111/ejn.15585. 34978115 PMC9306885

[psyp70003-bib-0034] Meissner, S. N. , M. Bächinger , S. Kikkert , et al. 2023. “Self‐Regulating Arousal via Pupil‐Based Biofeedback.” Nature Human Behaviour 1: 43–62. 10.1038/s41562-023-01729-z. PMC10810759 37904022

[psyp70003-bib-0035] Melnychuk, M. C. , P. M. Dockree , R. G. O'Connell , P. R. Murphy , J. H. Balsters , and I. H. Robertson . 2018. “Coupling of Respiration and Attention via the Locus Coeruleus: Effects of Meditation and Pranayama.” Psychophysiology 55, no. 9: e13091. 10.1111/psyp.13091. 29682753

[psyp70003-bib-0036] Melnychuk, M. C. , I. H. Robertson , E. R. G. Plini , and P. M. Dockree . 2021. “A Bridge Between the Breath and the Brain: Synchronization of Respiration, a Pupillometric Marker of the Locus Coeruleus, and an EEG Marker of Attentional Control State.” Brain Sciences 11, no. 10: 1324. 10.3390/brainsci11101324. 34679389 PMC8534189

[psyp70003-bib-0037] Morgado‐Valle, C. , J. Fernandez‐Ruiz , L. Lopez‐Meraz , and L. Beltran‐Parrazal . 2015. “Substitution of Extracellular Ca2+ by Sr2+ Prolongs Inspiratory Burst in Pre‐Bötzinger Complex Inspiratory Neurons.” Journal of Neurophysiology 113, no. 4: 1175–1183. 10.1152/jn.00705.2014. 25429120

[psyp70003-bib-0038] Muñoz‐Ortiz, J. , E. Muñoz‐Ortiz , L. López‐Meraz , L. Beltran‐Parrazal , and C. Morgado‐Valle . 2019. “The Pre‐Bötzinger Complex: Generation and Modulation of Respiratory Rhythm.” Neurología 34, no. 7: 461–468. 10.1016/j.nrleng.2018.05.006. 27443242

[psyp70003-bib-0039] Murata, A. , and H. Iwase . 2000. “Evaluation of Mental Workload by Variability of Pupil Area.” IEICE Transactions on Information and Systems E83: 1187–1190.

[psyp70003-bib-0040] Murphy, P. R. , R. G. O'Connell , M. O'Sullivan , I. H. Robertson , and J. H. Balsters . 2014. “Pupil Diameter Covaries With BOLD Activity in Human Locus Coeruleus.” Human Brain Mapping 35, no. 8: 4140–4154. 10.1002/hbm.22466. 24510607 PMC6869043

[psyp70003-bib-0041] Murphy, P. R. , I. H. Robertson , J. H. Balsters , and R. G. O'Connell . 2011. “Pupillometry and P3 Index the Locus Coeruleus‐Noradrenergic Arousal Function in Humans.” Psychophysiology 48, no. 11: 1532–1543. 10.1111/j.1469-8986.2011.01226.x. 21762458

[psyp70003-bib-0042] Nakamura, N. H. , M. Fukunaga , T. Yamamoto , N. Sadato , and Y. Oku . 2022. “Respiration‐Timing‐Dependent Changes in Activation of Neural Substrates During Cognitive Processes. Cerebral Cortex.” Communications 3, no. 4: tgac038. 10.1093/texcom/tgac038. PMC9552779 36237849

[psyp70003-bib-0043] Noble, D. J. , and S. Hochman . 2019. “Hypothesis: Pulmonary Afferent Activity Patterns During Slow, Deep Breathing Contribute to the Neural Induction of Physiological Relaxation.” Frontiers in Physiology 10: 1176. 10.3389/fphys.2019.01176. 31572221 PMC6753868

[psyp70003-bib-0044] Parnandi, A. , and R. Gutierrez‐Osuna . 2013. “Contactless Measurement of Heart Rate Variability From Pupillary Fluctuations.” Humaine Association Conference on Affective Computing and Intelligent Interaction 2013: 191–196. 10.1109/ACII.2013.38.

[psyp70003-bib-0045] Perl, O. , A. Ravia , M. Rubinson , et al. 2019. “Human Non‐Olfactory Cognition Phase‐Locked With Inhalation.” Nature Human Behaviour 3, no. 5: 5. 10.1038/s41562-019-0556-z. 31089297

[psyp70003-bib-0046] Regnath, F. , and S. Mathôt . 2021. “Pupil Size Reflects Exploration and Exploitation in Visual Search (And It's Like Object‐Based Attention).” bioRxiv. 10.1101/2021.02.05.429946.

[psyp70003-bib-0047] Reimer, J. , M. J. McGinley , Y. Liu , et al. 2016. “Pupil Fluctuations Track Rapid Changes in Adrenergic and Cholinergic Activity in Cortex.” Nature Communications 7: 13289. 10.1038/ncomms13289. PMC5105162 27824036

[psyp70003-bib-0048] Samuels, E. R. , and E. Szabadi . 2008. “Functional Neuroanatomy of the Noradrenergic Locus Coeruleus: Its Roles in the Regulation of Arousal and Autonomic Function Part II: Physiological and Pharmacological Manipulations and Pathological Alterations of Locus Coeruleus Activity in Humans.” Current Neuropharmacology 6, no. 3: 254–285. 10.2174/157015908785777193. 19506724 PMC2687931

[psyp70003-bib-0049] Schaefer, M. , S. Edwards , F. Nordén , J. N. Lundström , and A. Arshamian . 2023. “Inconclusive Evidence That Breathing Shapes Pupil Dynamics in Humans: A Systematic Review.” Pflügers Archiv / European Journal of Physiology 475, no. 1: 119–137. 10.1007/s00424-022-02729-0. 35871662 PMC9816272

[psyp70003-bib-0050] Schaefer, M. , S. Mathôt , M. Lundqvist , J. N. Lundström , and A. Arshamian . 2024. “The Respiratory‐Pupillary Phase Effect: Pupils Size Is Smallest Around Inhalation Onset and Largest During Exhalation.” bioRxiv. 10.1101/2024.06.27.599713. PMC11908488 39981599

[psyp70003-bib-0051] Schelegle, E. S. 2003. “Functional Morphology and Physiology of Slowly Adapting Pulmonary Stretch Receptors.” Anatomical Record Part A: Discoveries in Molecular, Cellular, and Evolutionary Biology 270A, no. 1: 11–16. 10.1002/ar.a.10004. 12494485

[psyp70003-bib-0052] Schumann, A. , C. Andrack , and K.‐J. Bär . 2017. “Differences of Sympathetic and Parasympathetic Modulation in Major Depression.” Progress in Neuro‐Psychopharmacology and Biological Psychiatry 79: 324–331. 10.1016/j.pnpbp.2017.07.009. 28710030

[psyp70003-bib-0053] Schumann, A. , S. Kietzer , J. Ebel , and K. J. Bär . 2020. “Sympathetic and Parasympathetic Modulation of Pupillary Unrest.” Frontiers in Neuroscience 14: 178. 10.3389/fnins.2020.00178. 32218721 PMC7078331

[psyp70003-bib-0054] Schumann, A. , C. Kralisch , and K.‐J. Bär . 2015. “Spectral Decomposition of Pupillary Unrest Using Wavelet Entropy.” 2015 37th Annual International Conference of the IEEE Engineering in Medicine and Biology Society (EMBC), 6154–6157. 10.1109/EMBC.2015.7319797. 26737697

[psyp70003-bib-0055] Unsworth, N. , A. L. Miller , and S. Aghel . 2022. “Effort Mobilization and Lapses of Sustained Attention.” Cognitive, Affective, & Behavioral Neuroscience 22, no. 1: 42–56. 10.3758/s13415-021-00941-6. 34410617

[psyp70003-bib-0056] Unsworth, N. , and M. K. Robison . 2016. “Pupillary Correlates of Lapses of Sustained Attention.” Cognitive, Affective, & Behavioral Neuroscience 16, no. 4: 601–615. 10.3758/s13415-016-0417-4. 27038165

[psyp70003-bib-0057] Unsworth, N. , and M. K. Robison . 2018. “Tracking Arousal State and Mind Wandering With Pupillometry.” Cognitive, Affective, & Behavioral Neuroscience 18, no. 4: 638–664. 10.3758/s13415-018-0594-4. 29654476

[psyp70003-bib-0058] van den Brink, R. L. , P. R. Murphy , and S. Nieuwenhuis . 2016. “Pupil Diameter Tracks Lapses of Attention.” PLoS One 11, no. 10: e0165274. 10.1371/journal.pone.0165274. 27768778 PMC5074493

[psyp70003-bib-0059] Yackle, K. , L. A. Schwarz , K. Kam , et al. 2017. “Breathing Control Center Neurons That Promote Arousal in Mice.” Science 355, no. 6332: 1411–1415. 10.1126/science.aai7984. 28360327 PMC5505554

[psyp70003-bib-0060] Yoshida, H. , K. Yana , F. Okuyama , and T. Tokoro . 1994. “Time‐Varying Properties of Respiratory Fluctuations in Pupil Diameter of Human Eyes.” Methods of Information in Medicine 33, no. 1: 46–48. 10.1055/s-0038-1634990. 8177078

[psyp70003-bib-0061] Zaccaro, A. , A. Piarulli , M. Laurino , et al. 2018. “How Breath‐Control Can Change Your Life: A Systematic Review on Psycho‐Physiological Correlates of Slow Breathing.” Frontiers in Human Neuroscience 12: 353. 10.3389/fnhum.2018.00353. 30245619 PMC6137615

[psyp70003-bib-0062] Zelano, C. , H. Jiang , G. Zhou , et al. 2016. “Nasal Respiration Entrains Human Limbic Oscillations and Modulates Cognitive Function.” Journal of Neuroscience 36, no. 49: 12448–12467. 10.1523/JNEUROSCI.2586-16.2016. 27927961 PMC5148230

