# Supplementary material for: Paced Breathing Associated With Pupil Diameter Oscillations at the Same Rate and Reduced Lapses in Attention
Source: Psychophysiology. 2025 Feb 4;62(2):e70003. doi: 10.1111/psyp.70003 (PMC11794674; doi:10.1111/psyp.70003)

**Appendix 1 – Manipulation checks and task validity**

**Experiment 1**

Six NIB and one IB participant(s) were excluded from the full cohort due to incomplete data collection (n = 59; NIB n = 32, IB n = 27). As discussed below, for the behavioural analysis, a further one participant from the NIB group and four participants were excluded from the IB group, resulting in NIB n = 31, IB n = 23.

**PACE Task - Sustained Attention Task – Both Groups**

Non-compliance to responding was evident in 2 IB participants, clicking 0% and 70% of the expected 340. They were both excluded from subsequent analyses (IB n = 25). Otherwise, participants responded >99% of expected clicks and actually tended to exceed 340, seen by the mean proportion of ‘clicks out of expected’: IB M = 1.05 ± 0.01, NIB M = 1.045 ± 0.01. Figure S1 shows this metric across the blocks. Testing for differences here showed that there was no significant effect of blocks, F(4) = 0.91, p = 0.46, nor group, F(1) = 0.98, p = 0.33, nor interaction, F(4) = 0.43, p = 0.79.

To assess the tasks accessibility subjectively, we asked participants ‘*Were you able to easily follow along with the tones using mouse clicks?’*. 44/57 participants (20 IB, 24 NIB) gave a solely positive response with regards to this. 7 participants (3 IB, 4 NIB) gave mixed responses, citing difficulty in tone discrimination or mixing up the left/right clicks. 6 participants (1 IB, 5 NIB) gave a majorly negative response, 3 zoning out from the beginning, 2 forgetting the left/right click order completely, 1 couldn’t tell the tones apart and 1 giving no reason.

**PACE Task - Auditory-Respiratory Entrainment – IB Group**

To assess whether the PACE task as effective as a paced breath guide for the IB group (IB n = 25), we analysed the respiratory phases at tone transition time to see if they were aligned and consistent. Rayleigh tests rejected the null hypothesis of uniformity for of respiratory phases at both LH and HL transitions for all IB participants, all p values < 0.001, indicating significant clustering. Resultant auditory-respiratory vector lengths ranged between 0.34 - 0.97 for LH; M = 0.86 ± 0.03 and 0.23 - 0.97 for HL; M = 0.81 ± 0.04 (Figure S2a). Two IB participants appeared to entrain relatively poorly, exhibiting low LH and HL vector lengths that were comparable to the NIB group (> 2 standard deviations less than group mean). They were therefore excluded from subsequent analyses as they could not be considered to have followed the paced breathing intervention sufficiently (IB n = 23). Without these participants, all IB auditory-respiratory vector lengths were > 0.58.

Examining the mean respiratory phase angles that the auditory-respiratory vector lengths tended towards, the IB group level pattern for entrainment shows that they already began inhaling or exhaling prior to tone transition points, as indicated by mean phase angles at these times: LH = -2.52 rad, HL = 0.91 rad, representing early inhale and early exhale respectively (Figure S2c). These angles differed across blocks 1, 3 and 5 as seen by Watson-Williams test: HL p < 0.001, LH p = 0.003, with block 3 (HL = 1.2 rad, LH = -2.3) showing the mean respiratory angle further into the inhale and exhale than in blocks 1 (HL = 0.84, LH = -2.6) and 5 (HL = 074, LH = -2.6).

Entrainment appeared to diminish over the course of the task, represented by a significant difference in auditory-respiratory vector lengths across blocks, decreasing for both LH, F(4) = 5.43, p < 0.001, and HL, F(4) = 9.58, p < 0.001.

IB group participants appeared to entrain more tightly with LH than HL transitions, with the task level mean LH vector length being significantly larger, t(22) = 3.93, p < 0.001. Differences appear to emerge later on in the task (Figure S2b).

See Figure 5 for the group average spectrogram of respiration over the task to note how the respiratory frequency changed over the task. It is additionally worth noting that participants’ resting respiration rate did not correlate significantly with any entrainment metric and thus the ability to follow the guided breath rates was likely independent of this.

**Auditory-Respiratory Entrainment - NIB**

We also checked for evidence of respiratory entrainment in the NIB group to see if this occurred without any explicit instruction to (NIB n = 32).

For a subjective assessment, we asked participants *‘How much attention did you pay to your breath during the study?’*. 7 NIB participants made reference to some degree of focus on the breath, 4 explicitly mentioning aligning the tones with the breath. 3 of those 4 participants were previously excluded for incomplete data and the remaining 1 was excluded from subsequent analyses based on this comment and relatively high auditory-respiratory vector lengths (M = 0.41; > 2 standard deviations above group mean) (NIB n = 31). The other 3/7 mentioned some other aspect of breath e.g., depth. Besides these notable exceptions, it appeared as though the NIB group was generally naive to the guided breath aspect. When asked: *‘What did you think the point of this study was?’* 8 NIB participants mentioned ‘breath’, but it was often in conjunction with ‘heart rate’ or ‘eye movements’, and no participant made any explicit reference to guided breathing.

Rayleigh tests showed 5/32 NIB participants with significant non-uniformity (p < 0.05) for LH only, 3/32 for HL only and 16/32 for both. Therefore, 24/32 NIB participants showed some significant degree of respiratory entrainment to the stimuli according to this metric. Auditory-respiratory vector lengths for the NIB group were: LH ranged 0.04 - 0.39, M = 0.17 ± 0.015; HL, 0.03 - 0.43, M = 0.17 ± 0.02. Nearly all of the mean phase angles for each NIB participant at the time of tone transitions lay between early-mid exhale to early inhale (Figure S1.2c). The mean phase angle for LH was slightly later in the exhale, 2.79 rad, than for HL, 2.08 rad, with a Watson-Williams test indicating a significant difference, F(1) = 6.81, p = 0.01.

There was a significant difference across blocks for HL vector lengths, F(4) = 3.96, p = 0.005 (Figure S2b), with post-hoc Holm tests showing significant decreases between blocks 1 and 3 and blocks 1 and 4 (both p < 0.01). There was no significant difference across blocks for LH vector lengths, F(4) = 1.60, p = 0.20. There was no significant difference between LH and HL vector lengths at the task level.

It is worth noting here that the spectrogram for NIB respiration (Figure 4) shows some activity at 0.1 Hz during block 1, and then the rest appears more random.

Despite showing evidence of respiratory entrainment to tones, i.e., significant Rayleigh test non-uniformity and small-medium respiratory vector lengths, the NIB group did this to a considerably lesser extent and with a different phase pattern when compared to the IB group. We believe that the entrainment was subtle enough to continue with the further analyses comparing the groups for an effect of instructed SPB.


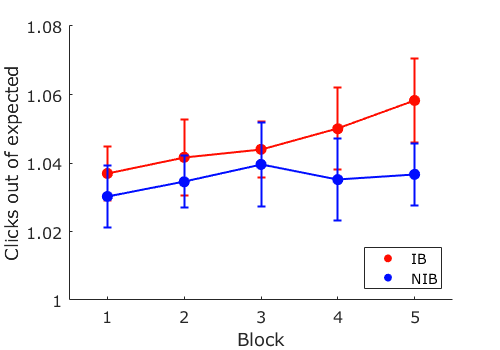


**Figure S1** – Exp. 1 -

Proportion of clicks made out of the total expected across blocks. Error bars represent the standard error of the mean.


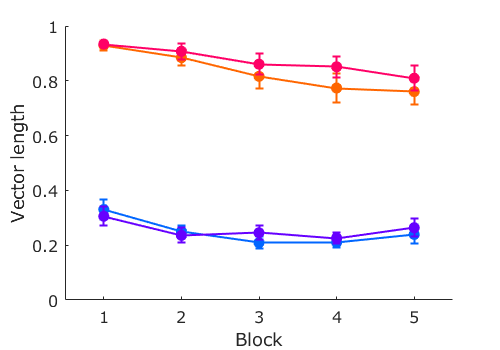

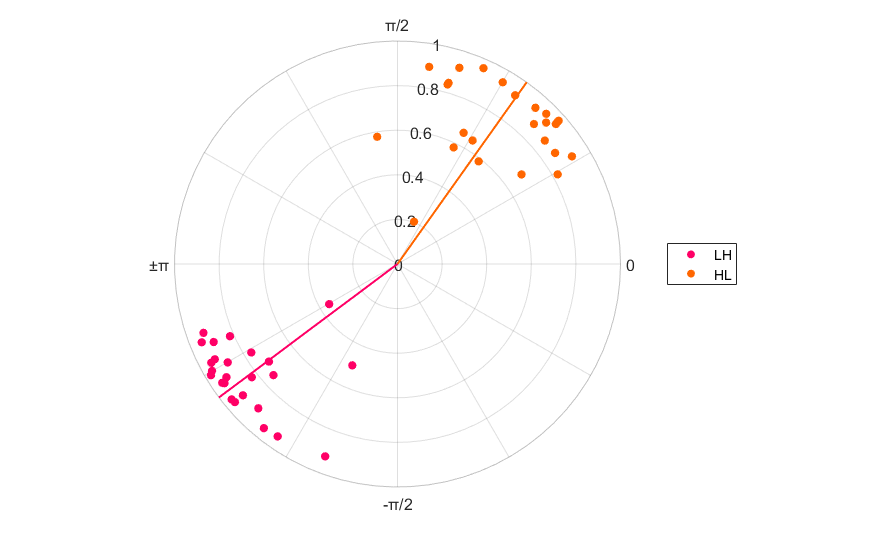

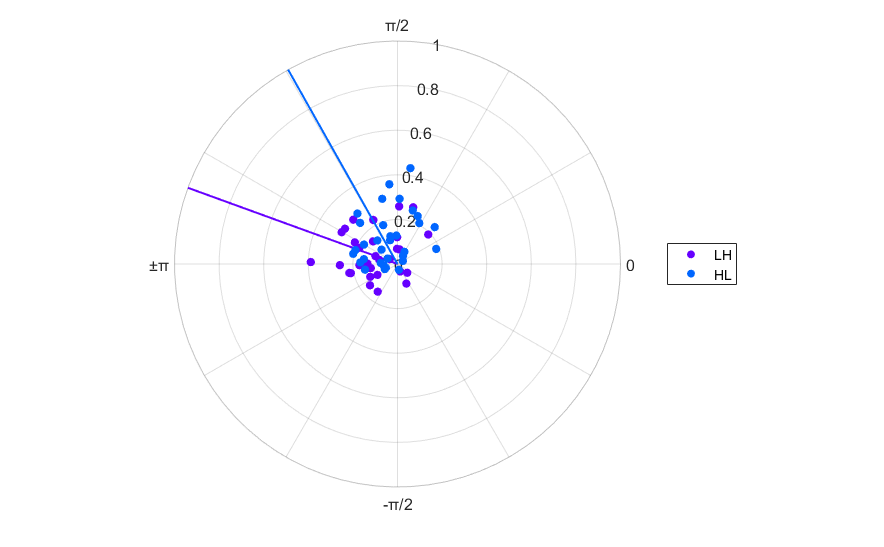


**Figure S2a** – Exp. 1 - Task level mean auditory-respiratory vector lengths, representing the degree of respiratory entrainment to tone transition times, HL and LH.


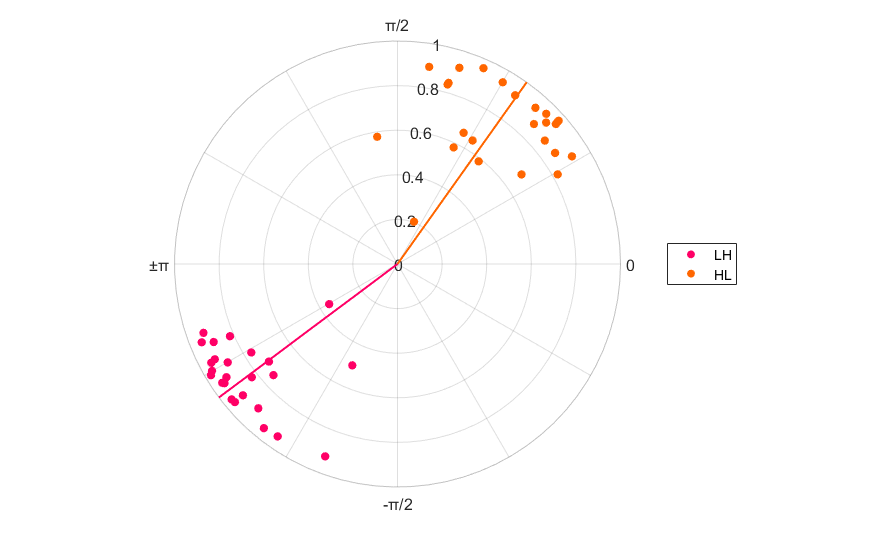

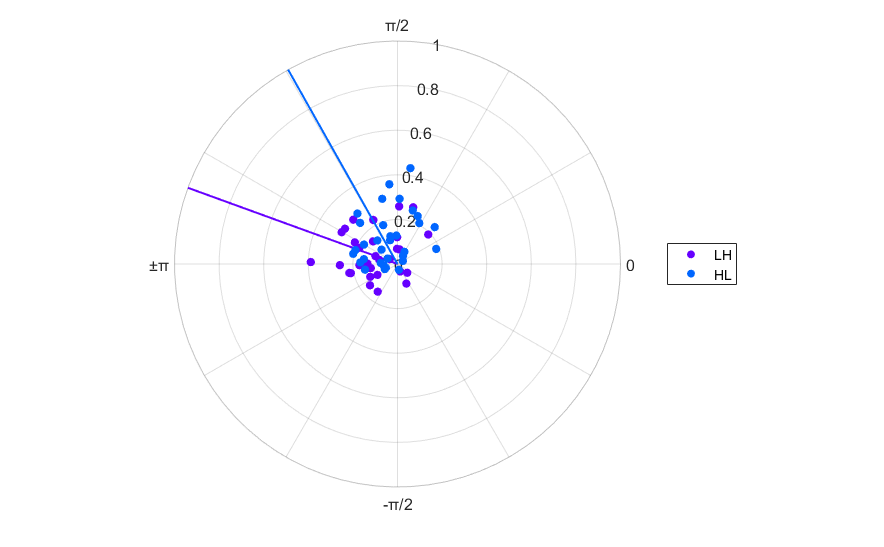


**Figure S2c** - Exp. 1 - Task level mean auditory-respiratory vector lengths (radial axis) and the mean phase angle of entrainment (angular axis) split by tone transition type. Lines indicate the mean phase angle for each group. Phase 0 rad is exhale onset and the breath follows anticlockwise.

**IB**

**NIB**

**Figure S2b** - Exp. 1 - Mean auditory-respiratory vector lengths at each block, split by tone transition type and group. Error bars represent the standard error of the mean.


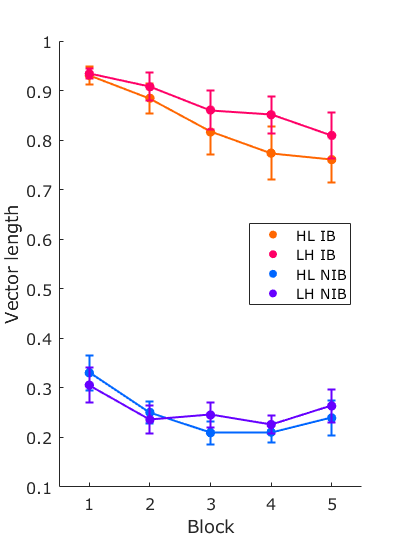

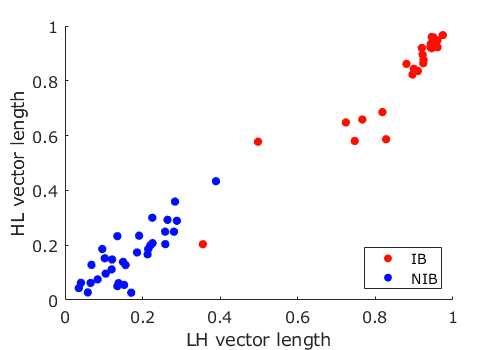


**Experiment 2**

Full cohort for Experiment 2 was n = 63, IB = 32, NIB = 31. As discussed below, for the behavioural analysis, nine IB participants and six NIB participants were excluded, resulting in NIB n = 25, IB n = 23.

**PACE Task - Sustained Attention Task – Both Groups**

Non-compliance to responding was evident in 1 IB participants, clicking 48% out of the expected 350 clicks. They were excluded from subsequent analyses (IB n = 31). Otherwise, participants responded >87% of expected clicks and actually tended to exceed 350, seen by the mean proportion of ‘clicks out of expected’: IB M = 1.00 ± 0.02, NIB M = 1.01 ± 0.01. Figure S3 shows this metric across the blocks. Testing for differences here showed that there a significant main effect of blocks, F(2) = 3.60, p = 0.03, but not group, F(1) = 0.42, p = 0.52, nor interaction, F(2) = 0.30, p = 0.74. The significant difference in blocks was found post-hoc as a reduction from block 1 to 2, Holm test p = 0.03 (adjusted for family of 3).

To assess the tasks accessibility subjectively, we asked participants ‘*Were you able to easily follow along with the tones using mouse clicks?’*. 31/63 participants (15 IB, 16 NIB) gave an overall positive response with regards to this. 27 participants (15 IB, 12 NIB) gave mixed responses, citing difficulty in tone discrimination or mixing up the left/right clicks, or feeling sleepy or distracted at points. 5 participants (3 IB, 2 NIB) gave a majorly negative response, all citing difficulty in tone discrimination.

**PACE Task - Auditory-Respiratory Entrainment – IB Group**

IB group n = 31. Rayleigh tests rejected the null hypothesis of uniformity for of respiratory phases at both LH and HL transitions for all IB participants, all p values < 0.001, indicating significant clustering. Resultant auditory-respiratory vector lengths ranged between 0.45 - 0.97 for LH; M = 0.86 ± 0.02 and 0.21 - 0.99 for HL; M = 0.79 ± 0.03 (Figure S4a). Eight IB participants appeared to entrain relatively poorly, exhibiting LH and HL vector lengths that were at least 2 standard deviations below the group mean. They were therefore excluded from subsequent analyses as they could not be considered to have followed the paced breathing intervention sufficiently (IB n = 23). Without these participants, all IB auditory-respiratory vector lengths were > 0.75.

Examining the mean respiratory phase angles that the auditory-respiratory vector lengths tended towards, the IB group level pattern for entrainment shows that they already began inhaling or exhaling prior to tone transition points, as indicated by mean phase angles at these times: LH = -2.43 rad, HL = 1.00 rad, representing early inhale and early exhale respectively (Figure S4c). These angles differed across blocks 1 and 3 as seen by Watson-Williams test: LH p < 0.001, HL p = 0.002, with block 3 (LH = -2.30, HL = 1.22 rad) showing the mean respiratory angle further into the inhale and exhale than in block 1 (LH = -2.54 HL = 0.87).

With regards to auditory-respiratory vector lengths over blocks 1,2 and 3, there was no significant difference for LH, F(2) = 1.94, p = 0.16, but there was for HL, F(2) = 7.70, p = 0.005, decreasing over the blocks.

IB group participants appeared to entrain more tightly with LH than HL transitions, with the task level mean LH vector length being significantly larger, t(22) = 3.47, p = 0.002. Differences appear to emerge later on in the task (Figure S4b).

See Figure 10 for the group average spectrogram of respiration over the task to note how the respiratory frequency changed over the task. It is additionally worth noting that participants’ resting respiration rate did not correlate significantly with any entrainment metric and thus the ability to follow the guided breath rates was likely independent of this.

**Auditory-Respiratory Entrainment - NIB**

We also checked for evidence of respiratory entrainment in the NIB group to see if this occurred without any explicit instruction to (NIB n = 31).

For a subjective assessment, we asked participants *‘How much attention did you pay to your breath during the study?’*. 13 NIB participants made reference to some degree of focus on the breath. 2 explicitly mentioned aligning the tones with the breath, however, their actual auditory-respiratory vector lengths were very close to the mean of the group and were not excluded. The other participants mentioned some other aspect of breath e.g., depth, nasal/oral, or in an unspecified manner. Besides these notable exceptions, it appeared as though the NIB group was generally naive to the guided breath aspect.

When asked: *‘What did you think the point of this study was?’* 4 NIB participants mentioned ‘breath’, but this was in conjunction with ‘heart rate’ or ‘eye movements’, and no participant made any explicit reference to guided breathing.

Rayleigh tests showed 22/31 NIB participants with significant non-uniformity (p < 0.05) for LH only, 24/31 for HL only and 22/31 for both. Therefore, 24/31 NIB participants showed some significant degree of respiratory entrainment to the stimuli according to this metric.

Auditory-respiratory vector lengths for the NIB group were: LH ranged 0.02 - 0.83, M = 0.22 ± 0.03; HL, 0.05 - 0.87, M = 0.22 ± 0.03. Six NIB participants were deemed to have high vector lengths, being more than 2 standard deviations above the group mean, and were excluded from subsequent analyses (NIB n = 25). Without these participants, all NIB auditory-respiratory vector lengths were < 0.32.

Nearly all of the mean phase angles for each NIB participant at the time of tone transitions lay between early-mid exhale to early inhale (Figure S4c). The mean phase angle for LH was slightly later in the exhale, 2.68 rad, than for HL, 2.09 rad, with a Watson-Williams test indicating a significant difference, F(1) = 7.00, p = 0.01.

There was no significant difference across blocks 1, 2 and 3, for LH vector lengths, F(2) = 0.79, p = 0.46. There was no significant difference across blocks for HL vector lengths, F(2) = 2.94, p = 0.06, though there is a trend for a sharp decrease in block 3 (Figure S4b).

Despite showing evidence of respiratory entrainment to tones, i.e., significant Rayleigh test non-uniformity and small-medium respiratory vector lengths, the NIB group did this to a considerably lesser extent and with a different phase pattern when compared to the IB group. We believe that the entrainment was subtle enough to continue with the further analyses comparing the groups for an effect of instructed SPB.


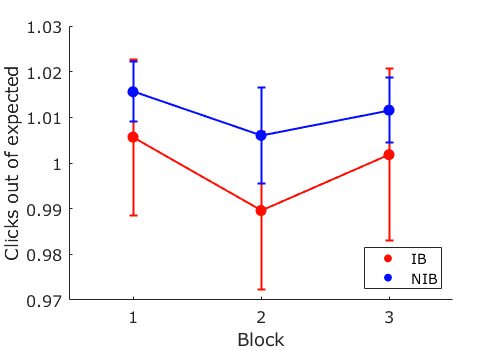


**Figure S3** – Exp. 2 -

Proportion of clicks made out of the total expected across blocks. Error bars represent the standard error of the mean.


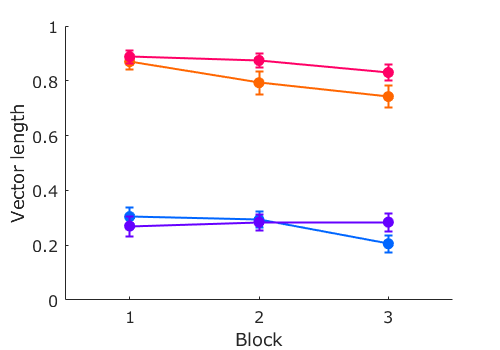

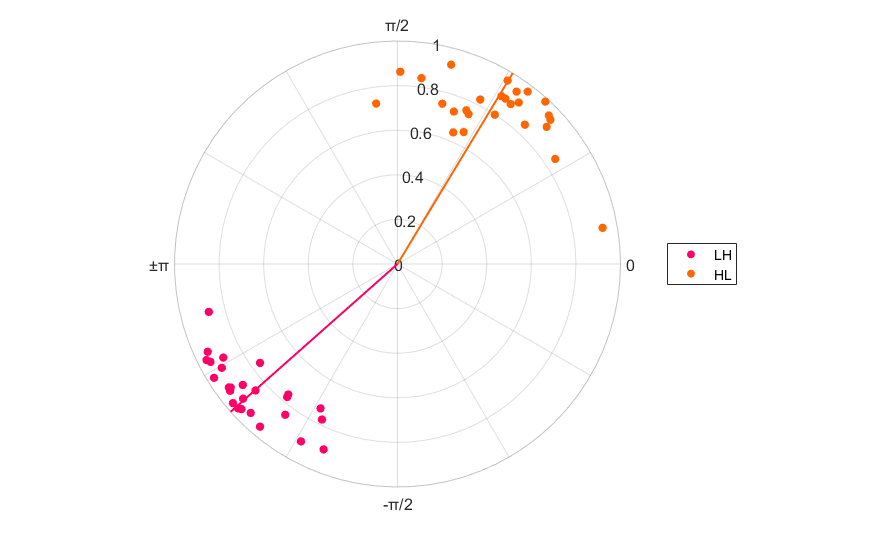

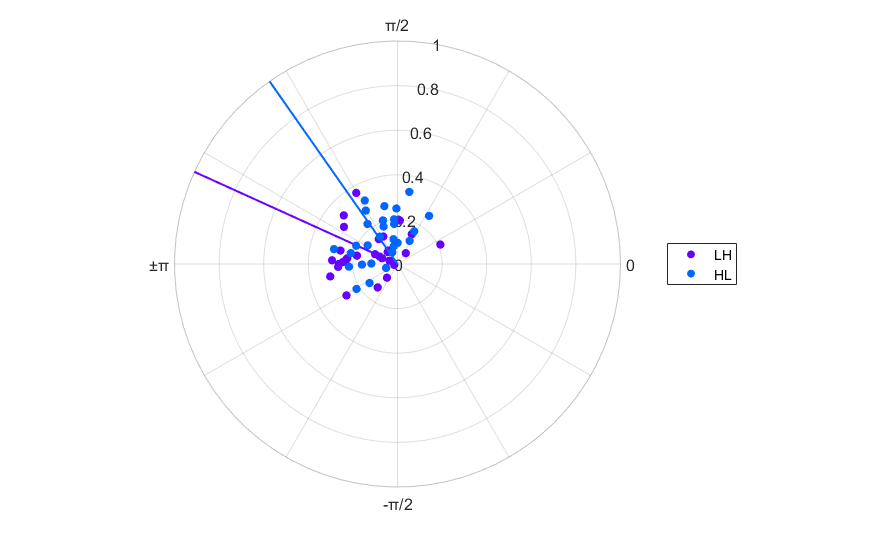


Figure S4a - Exp. 2 - Task level mean vector lengths, representing the degree of respiratory entrainment to tone transition times, HL and LH.


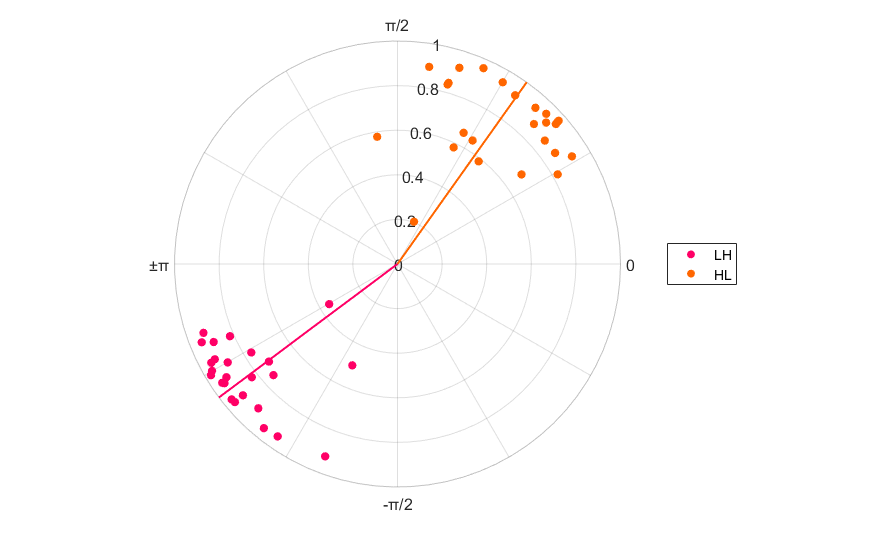

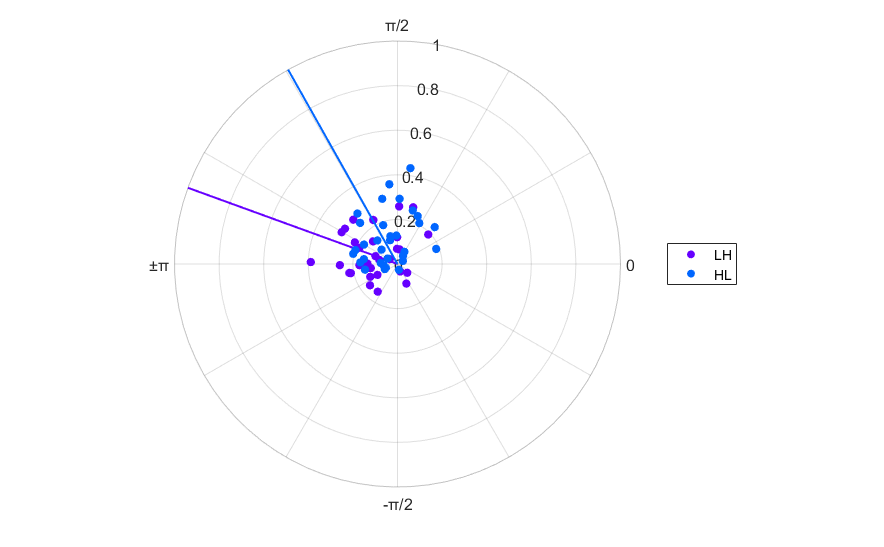


Figure S4c - Exp. 2 - Task level mean vector lengths (radial axis) and the mean phase angle of entrainment (angular axis) split by tone transition type. Lines indicate the mean phase angle for each group. Phase 0 rad is exhale onset and the breath follows anticlockwise.

**IB**

**NIB**

Figure S4b - Exp. 2 - Mean vector lengths at each block, split by tone transition type and group. Error bars represent the standard error of the mean.


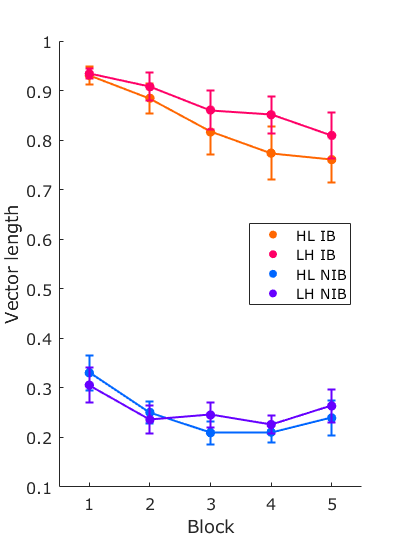

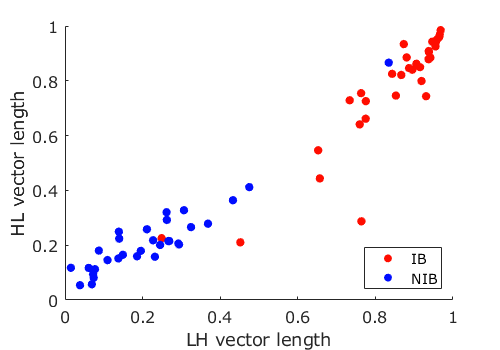

Supplement: Supplementary file 1 — Appendix S1. [file PSYP-62-e70003-s001.docx]
